# ALS mutations do not alter perineuronal net formation in human stem cell-derived motor neurons

**DOI:** 10.1038/s42003-026-10244-6

**Published:** 2026-05-19

**Authors:** Caoimhe Kerins, Ivo Lieberam, Eileen Gentleman

**Affiliations:** 1 Centre for Craniofacial and Regenerative Biology, King’s College London, London, UK; 2 Centre for Gene Therapy and Regenerative Medicine, King’s College London, London, UK; 3 Centre for Developmental Neuroscience, King’s College London, London, UK; 4 Department of Biomedical Sciences, University of Lausanne, Lausanne, Switzerland

**Keywords:** Pluripotent stem cells, Neurological disorders

## Abstract

Perineuronal nets (PNNs) are extracellular matrix structures that stabilise synaptic inputs and play a role in regulating neuronal plasticity. Although PNN dysregulation is observed in several neurological disorders, their relevance to amyotrophic lateral sclerosis (ALS) remains unclear. In particular, the extent to which PNN alterations reported in ALS animal models are motor neuron (MN)-intrinsic is unknown. We investigated whether human pluripotent stem cell-derived MNs form PNN-like structures in vitro, and whether ALS-associated mutations alter this process. We show that PNN-like structures containing hyaluronan, tenascin-R, and aggrecan form in in vitro co-cultures of iPSC-derived MNs and astrocytes, and that their formation and gene expression were not altered by ALS mutations. To explore whether PNN dysregulation reflects contributions from other cell types or selective MN vulnerability, we conducted meta-analyses of transcriptomic datasets from pluripotent stem cell-derived astrocytes carrying ALS-associated mutations, as well as datasets comparing MN populations with differential susceptibility to ALS. These analyses revealed no consistent differences in PNN-related gene expression within human stem cell-derived MNs. In contrast, transcriptomic analyses of human post-mortem ALS tissues revealed dysregulation of PNN-related genes, including core PNN components. Taken together, these findings indicate that PNN-related alterations described in ALS animal models are not reproduced by ALS-associated mutations in MNs alone, and instead point to a role for additional cellular components within the central nervous system.

## Abstract (web summary)

Human stem cell-derived motor neurons form perineuronal net-like extracellular matrix structures in vitro, but ALS-associated mutations do not alter their formation, suggesting additional factors beyond motor neurons may contribute to changes seen in ALS.

## Introduction

Amyotrophic lateral sclerosis (ALS) is a degenerative neuromuscular disease characterised by death of motor neurons (MNs) and resultant muscle paralysis. There is currently no cure for ALS with most patients dying of neuromuscular respiratory failure within 2-5 years of diagnosis^1^. 80-90% of ALS cases are sporadic and are usually caused by an interplay of environmental and genetic factors^2^. The remaining cases have known heritable causes, and are predominantly associated with a single dominant mutation in one of ~30 known ALS-linked genes^3^, including chromosome 9 open reading frame 72 (C9orf72), superoxide dismutase 1 (SOD1), transactive response DNA binding protein 43 (TDP-43) and fused in sarcoma (FUS)^4^. Several genetic causes and risk factors, including biological sex and age, have been associated with ALS^2^. Moreover, multiple molecular changes have been noted in MNs in ALS models, including oxidative stress, excitotoxicity, neuroinflammation, neuronal hyperexcitation, and axonopathy^1^. Differing explanations have been proposed to explain MN death in ALS. For example, hypotheses commonly labelled “dying forward” and “dying back” propose alternative initiation sites for MN degeneration in ALS^5^. However, both may occur simultaneously or sequentially, and the key mechanism(s) that drive ALS pathology remain elusive. Adding to this complexity, MNs are not uniformly affected in ALS. For example, fast-twitch α-MNs are highly susceptible to cell death, whereas other MNs, such as those in the oculomotor nucleus, remain relatively preserved, even at advanced disease stages^6,7^. These uncertainties underscore the need to consider not only cell-intrinsic mechanisms but also extracellular influences that may shape MN vulnerability in ALS.

Three major extracellular structures make up the extracellular matrix (ECM) of the central nervous system (CNS): the basement membrane, perineuronal nets (PNNs), and the interstitial matrix. PNNs directly surround the cell body, proximal dendrites and axon initial segment of some neurons, including many interneurons and MNs^8,9^. PNN components are secreted by neurons, oligodendrocytes, microglia, and astrocytes^10^. They consist of a hyaluronan backbone and multiple proteoglycans, including aggrecan, versican, brevican, and other linker proteins, such as tenascin-R. In rats, up to 80% of spinal MNs have PNNs, and 90% of α-MNs are positive for aggrecan^11^. Components of PNNs are liable to degradation by a host of catabolic enzymes, including matrix metalloproteinases (MMPs) and members of the disintegrin and metalloproteinase with thrombospondin motifs (ADAMTS) family^12^. Various functions have been associated with PNNs, including protection of neurons against oxidative stress, particularly iron-induced oxidative stress^13^, regulation of memory and plasticity^14–16^, and control of neuronal excitability^17,18^. Alterations to PNNs have been reported in a range of neurological diseases such as Alzheimer’s disease, schizophrenia, multiple sclerosis and epilepsy^18–20^.

Several studies have suggested potential links between PNNs and ALS pathology. For example, both TDP-43 Q331K and SOD1 G93A mice show significant reductions in PNNs around α-MNs at disease onset and mid-stage compared to controls^21,22^. Similarly, transgenic rats harbouring a His46Arg mutation in the SOD1 gene demonstrate progressive loss of PNNs around susceptible MNs in the ventral horns of the lumbar spinal cord^23^. Additional work suggests that PNN components influence excitatory balance, alterations of which are a pathological hallmark of ALS. For example, depletion of hyaluronan synthase 3 (*Has3*), which is expressed in PNN-bearing MNs in the spinal cord^8^, results in an epileptic phenotype^24^. Excitatory synaptic transmission is also increased in tenascin-R-deficient mice^25^, which also exhibit motor coordination deficits^26,27^. Other studies have implicated matrix-remodelling enzymes. For example, while MMP9 is expressed by the most vulnerable MNs to cell death in ALS, it is absent in areas that are resistant to disease^28^. Moreover, reducing MMP9 function resulted in SOD1 G93A mice retaining 75% innervation in the normally vulnerable tibialis anterior muscle compared to controls^28^. Evidence in humans is more limited, but post-mortem studies on sporadic ALS patients have identified increases in hyaluronan binding protein 2 and decreases in tenascin-R in their cerebral spinal fluid^29^.

Taken together, these data suggest that maintenance of normal PNNs may help preserve MN function, and that their loss could contribute to disease-relevant phenotypes observed in ALS models. However, testing this hypothesis remains challenging due to key gaps in our understanding of PNNs, particularly in humans. Indeed, there is scant evidence on the extent to which human CNS cells (such as MNs) form PNNs, if ALS-associated mutations affect PNN production by MNs, and whether PNNs are altered in humans with ALS. Here, we show that human pluripotent stem cell-derived MNs form PNN-like structures in vitro, and that ALS mutations do not alter their formation or their expression of PNN-related genes. Similarly, a meta-analysis failed to detect differential expression of PNN-related genes when comparing resistant and vulnerable MN populations or mutant to control human induced pluripotent stem cell (hiPSC)-derived astrocytes. However, a re-analysis of post-mortem transcriptomic data revealed altered expression of PNN components in ALS tissues compared to healthy controls. These findings suggest that PNN-related alterations described in ALS animal models are not reproduced by ALS-associated mutations in MNs alone, and may instead reflect contributions from additional cellular components or factors not captured in 2D in vitro systems.

## Results

### Human pluripotent stem cell-derived MNs form PNNs in vitro

**Fig. 1:**
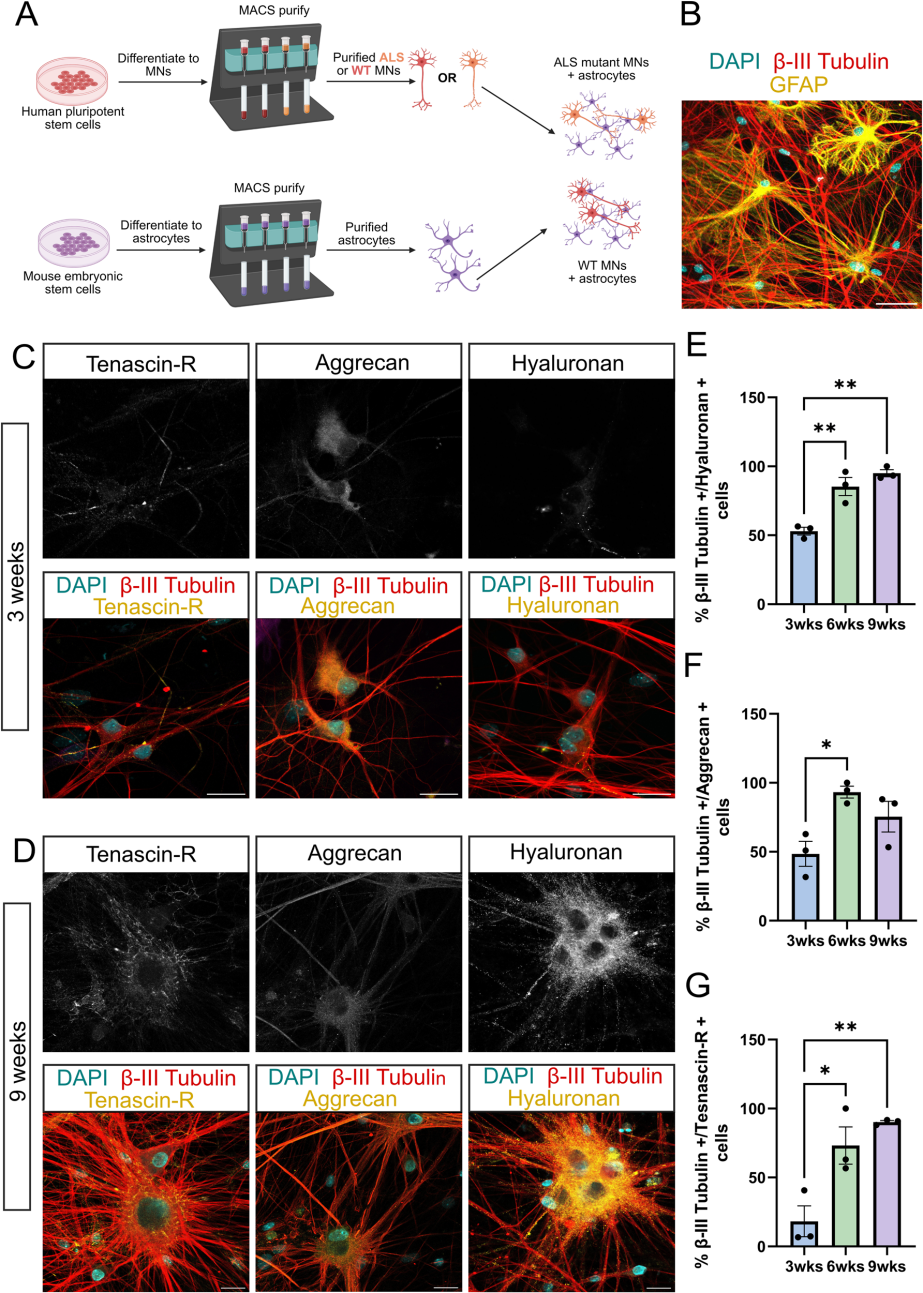
Human pluripotent stem cell-derived MNs form PNNs in vitro. PNN-like structures are deposited in co-cultures of WT human ESC-derived MNs and astrocytes. **A** Schematic overview of co-culture system. mESC were differentiated into astrocytes by directed differentiation and human WT ESC (H9) into MNs. At the end of differentiation, MACS was used to purify astrocyte and MN populations. Astrocytes were plated 1 week before MNs. Co-cultures were then maintained for either 3, 6 or 9 weeks. Created with BioRender. **B** Representative confocal microscopy image of immunostaining illustrating MNs (β-III tubulin) and astrocytes (GFAP) after 3 weeks in co-culture. Representative confocal microscopy images showing (**C**) 3-week and (**D**) 9-week staining of PNN components aggrecan, tenascin-R and hyaluronan in co-cultures of MNs and astrocytes. Scale bar = 25 µm. *N* = 3. Quantification of PNN-like structures in co-cultures of MNs and astrocytes at 3, 6 and 9 weeks. Quantification shows the percent of MNs that are double positive for β-III tubulin and PNN component (**E**) hyaluronan, (**F**) aggrecan, or **G**) tenascin-R. *N* = 3 differentiations with 5 images/*N*. *P < 0.05, **P < 0.01; ordinary, one-way ANOVA, with Tukey’s multiple comparison tests. Details of statistical tests are in Table S1.

Previous work has shown that primary cultures of rodent neurons and glial cells produce PNN-like structures in vitro^30–33^. However, there are currently no studies that report PNN formation by human pluripotent stem cell-derived CNS cells. Therefore, we first established a 2D model consisting of human pluripotent stem cell-derived MNs co-cultured with mouse embryonic stem cell (ESC)-derived astrocytes (Fig. 1A). Co-culture with astrocytes is a well-established technique for supporting MN cultures in vitro^34^, and for hiPSC-derived cultures, has been shown to promote neuronal maturation^35,36^. Our protocol relies on parallel directed differentiation of human ESC/iPSC to MNs^37^ and mouse ESC to astrocytes^38,39^. To generate co-cultures, we enriched each population using magnetic activated cell sorting (MACS): human MNs were isolated from Hb9::CD14-engineered hESCs/hiPSCs, and mouse astrocytes from GFAP::CD14-expressing ESCs^38–40^. MACS purification yielded MN enrichments exceeding 90%, and neuronal identity and maturation were validated using established markers (Figure S1). These co-cultures stained positively for Islet-1 and Glial Fibrillary Acidic Protein (GFAP), confirming the presence of both cell types (Fig. 1B and S1). For downstream imaging analyses, β-III tubulin was used to visualise neuronal soma and processes, enabling assessment of PNN-like structures surrounding both cell bodies and neuronal projections.

After 3 weeks, immunostaining confirmed that co-cultures were positive for PNN components hyaluronan and aggrecan, which have been shown to be necessary for the formation of a PNN-like matrix in vitro^41^; and tenascin-R, which plays essential roles in clustering aggrecan, stabilising nets and promoting the expression of brevican, neurocan and hyaluronan^42^ (Fig. 1C). Cultures also stained positively with Wisteria Floribunda Agglutin (WFA) (Figure S2). WFA is a lectin classically used to identify PNNs by binding to an unknown motif in chondroitin sulfate glycosaminoglycan chains. PNN-like structure deposition was not evident at earlier time points (Figure S3). After 9 weeks of co-culture, further accumulation of PNN components was evident (Fig. 1D), and there was a striking qualitative difference in the intensity of staining for PNN components in co-cultures after 9 weeks compared to 3 weeks. Cultures continued to stain positively with WFA, which was diffuse in contrast to PNN component aggrecan, which strongly localised with MNs (Figure S4). At 3 weeks, tenascin-R generally deposited around neurites, aggrecan was mostly around cell somata, and hyaluronan appeared punctate and spread around both cell somata and neurites. At later time points, quantification of immunostaining showed increased fractions of hyaluronan, tenascin-R and aggrecan positive cells that were also positive for β-III tubulin (Fig. 1E–G, Table S1, and Supplementary Code 1). After 9 weeks, tenascin-R was observed in a linearised/striped pattern around the cell somata of MNs and neurites. Aggrecan was again associated with cell somata as well as neurites, while hyaluronan maintained its punctate appearance around MN somata and neurites. PNN-like structures were concentrated around MNs and not astrocytes, which were found at the base of co-cultures (Figure S5). Thus, human pluripotent stem cell-derived MNs form PNN-like structures in vitro that robustly stain for hyaluronan, aggrecan and tenascin-R after 6 to 9 weeks in co-culture with astrocytes.

### ALS mutations do not impact PNN formation by human pluripotent stem cell-derived MNs

**Fig. 2:**
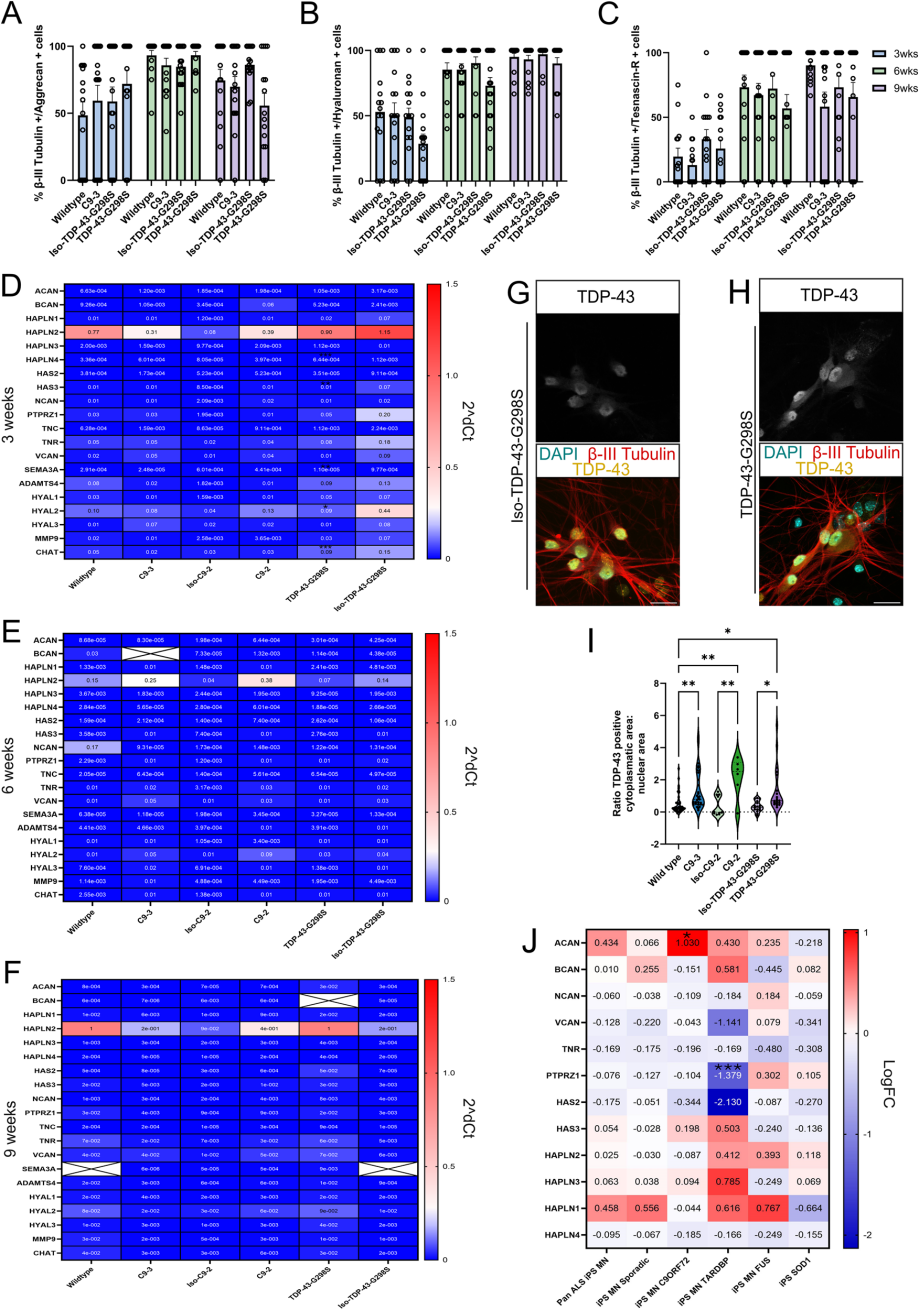
ALS mutations do not impact PNN formation by human pluripotent stem cell-derived MNs. Quantification of PNN-like structures in co-cultures of MNs and astrocytes at 3, 6 and 9 weeks. MNs were either WT (H9), C9orf72 mutant (C9-3), TARDBP mutant (TDP-43) or isogenic control TARDBP (Iso-TDP-43). Quantification shows the percent of MNs (β-III tubulin + ) that were also positively stained for PNN component (**A**) aggrecan, (**B**) hyaluronan, or (**C**) tenascin-R. *N* = 3 differentiations; 5 images/*N*. Relative gene expression for a range of PNN components and catabolic regulators of PNNs was performed using human-specific primers on co-cultures of MNs and astrocytes after (**D**) 3, (**E**) 6, and (**F**) 9 weeks in culture. 2^∆Ct^ values are relative to housekeeping genes (*GAPDH* or *ACTB*), and significance is indicated when there was a change in gene expression relative to the mutant line’s control (except for C9-3, which was compared to wildtype, Iso-C9-2 and Iso-TDP-43-G298S). Undetected genes are indicated by crossed-out boxes. *N* = 3. Representative confocal microscopy images of TDP-43 staining in (**G**) TDP-43 G298S isogenic controls and (**H**) TDP-43 G298S mutants in co-cultures. *N* = 3. **I** Quantification of TDP-43 cytoplasmic/nuclear localisation. *P < 0.05, **P < 0.01, ordinary one-way ANOVA. **J** Log fold change (LogFC) expression levels of genes encoding various PNN components generated from a meta-analysis comparing MNs derived from iPSC ALS mutant compared to control lines. ***P < 0.001.

Following confirmation that PNN-like structures were formed in in vitro co-cultures of MNs and astrocytes, we proceeded to form MNs from TDP-43 G298S and C9orf72 (C9-3) ALS mutant and isogenic control human iPSC lines (see Methods for detailed descriptions of the lines) using the same co-culture strategy outlined in Fig. 1A. Consistent with our observations in wild-type human ESCs, MNs formed from mutant and control iPSC lines all formed PNN-like structures that stained positively for hyaluronan, aggrecan, and tenascin-R (Figure S6). We then analysed these images to localise MNs (β-III tubulin-positive soma and projections) with positive staining for PNN components. We found that the fraction of MNs that were double positive for β-III tubulin and either hyaluronan, aggrecan or tenascin-R increased over the 9-week culture (Fig. 2A–C and Table S2). However, we could detect no significant differences in the number of double-positive MNs between any ALS mutant cell line and its control at any time point. Therefore, we conclude that TDP-43 G298S and C9-3 ALS mutations do not detectably affect the formation of aggrecan-, tenascin-R- and hyaluronan-containing PNN-like structures by human pluripotent stem cell-derived MNs in vitro.

Although immunostaining failed to show significant differences in PNN-like structure formation between MNs derived from TDP-43 G298S and C9-3 mutant and control lines, quantification of fluorescence images can be challenging as differences in the deposition of ECM components may be difficult to capture, even in extended cultures. Moreover, we only stained a limited number of components of PNNs. Therefore, we next aimed to expand our assessment of PNNs in co-cultures by performing qPCR on a panel of genes, including core PNN (*e.g*. versican *VCAN*, brevican *BCAN*, phosphacan *PTPRZ1*, *etc*.), and associated PNN regulatory genes (*e.g*. catabolic enzymes such as *MMP9*). We cultured MNs derived from iPSC with mutations in TDP-43 G298S, C9orf72 (C9-2), C9-3, and their controls (iso-TDP-43, iso-C9-2, and WT, respectively) and performed qPCR after 3, 6 and 9 weeks in culture using human-specific primers. We then plotted 2^∆Ct^ values for each gene, comparing expression in each mutant cell line to expression in its respective isogenic control line (or all control lines for C9-3) (Fig. 2D–F and Table S3).

Using this approach, we found no significant differences in the expression of any genes when comparing C9-2 and C9-3 mutants to control lines at any time point. We did identify significant increases in expression of a handful of genes including *HAS3*, *HAPLN4*, *HYAL2* and *SEM3A* after 3 weeks when comparing MNs derived from TDP-43 G298S mutants to their isogenic control. Nevertheless, we also found increased expression of choline acetyl transferase (*CHAT*), a MN maturation marker^37^, in TDP-43 G298S MNs relative to isogenic controls, suggesting that increased PNN gene expression in TDP-43 G298S mutants might be attributable to faster maturation in these cells^39^. This interpretation was supported by a lack of differential regulation between these conditions at weeks 6 and 9. The lack of differential regulation between mutant and isogenic control lines could not be attributed to a loss of phenotype in culture, as mutant lines showed significantly higher cytoplasmic TDP-43 localisation relative to their respective isogenic controls after 6 weeks in culture (Fig. 2G–I and Supplementary Code 2). Therefore, we conclude that familial ALS mutations tested here do not affect the transcription of genes that form PNNs in iPSC-derived MNs cultured in vitro.

As we had only worked with a limited number of ALS mutant lines, we next aimed to apply our approach more broadly by screening the expression of genes encoding PNN components in additional ALS mutant lines by analysing data from a previously published meta-analysis^43^. Ziff et al. had analysed a range of transcriptomic datasets from ALS mutant iPSC-derived MNs, including sporadic cases and mutations in C9orf72, FUS, SOD1, and TARDBP. We generated a list of gene ontology (GO) terms for PNNs and calculated log fold change (LogFC) values and adjusted p-values comparing expression in mutant lines to that in MNs generated from control iPSC lines, which we then plotted as heat maps (Fig. 2J). However, despite expanding the number of ALS-associated mutations that we analysed, we could not find strong evidence for consistent differential expression of genes associated with the formation of PNN components in mutants compared to controls. We did find that the gene for aggrecan (*ACAN*) was significantly upregulated in C9orf72 iPSC-derived MNs and that *PTPRZ1* was significantly downregulated in TARDBP mutant iPSC-derived MNs compared to MNs derived from control iPSC lines. However, given that these were the only significantly differentially regulated genes across this large dataset, it seems unlikely that there is a consistent intrinsic differential expression of PNN components in iPSC-derived MNs bearing ALS mutations.

### Altered expression of PNN components in human ALS post-mortem tissue is not captured by in vitro models

**Fig. 3:**
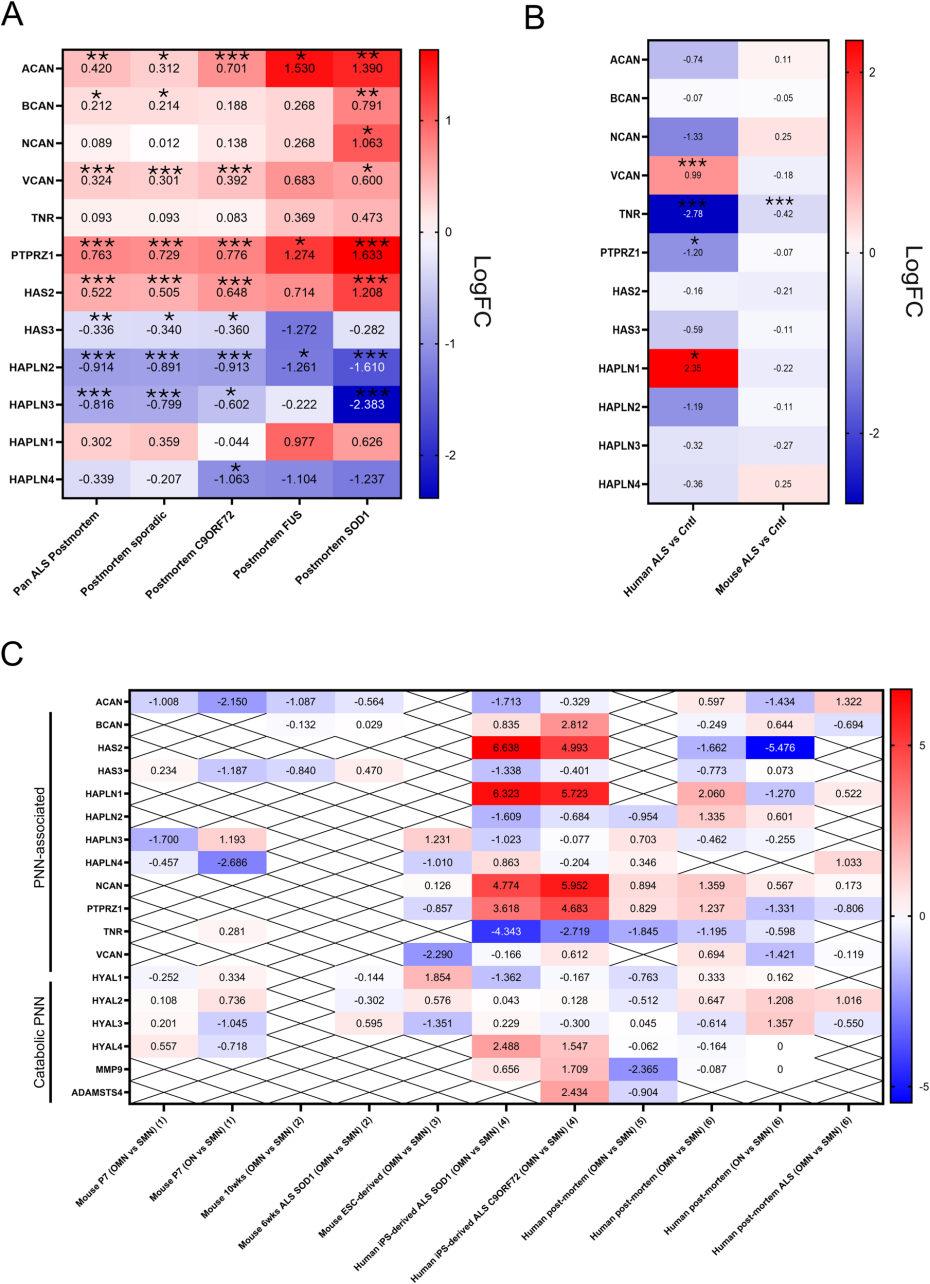
Altered expression of PNN components in human ALS post-mortem tissue is not captured by in vitro models. **A** PNN expression in human post-mortem ALS tissues. A) Log fold change (LogFC) values of ALS mutant human post-mortem tissues for PNN component visualised as a heatmap. The relevant ALS mutation is indicated on x-axis. **B** Log fold change (LogFC) values of ALS mutant human or mouse astrocytes for PNN-related GO term visualised as a heatmap. Data acquired from Ziff et al. (2022). **C** Expression of PNN and PNN-associated genes comparing resistant to susceptible tissues in healthy and ALS tissues and ALS cell models. Heatmaps show LogFC. Genes not expressed at detectable levels or that had multiple hits are indicated by an X through the relevant box. OMN = oculomotor neuron, ON = Onuf’s nucleus, SMN = spinal motor neurons. Each dataset is labelled by time point, model, comparison type (OMN vs SMN or ON vs SMN) and dataset origin (see Table S4). *P* adjusted values, * < 0.05, ** < 0.01, *** < 0.001, ****p < 0.0001.

Although our in vitro model allowed us to create PNN-like structures from human cells, MNs containing ALS-associated mutations failed to show evidence of the loss of PNNs that have been reported in animal models at either the protein or transcriptomic levels. However, our in vitro cultures are reductionist and fail to incorporate many in vivo complexities, including the 3D environment, blood flow, and contributions from other neuronal and glial cell types. Therefore, we next assessed transcriptomic data captured from post-mortem human ALS tissue samples. Using bulk RNAseq data gathered in the same meta-analysis by Ziff et al.^43^, which drew on data from the New York Genome Center’s ALS Cohort, including 214 ALS post-mortem spinal cord and 57 control samples, we assessed the expression of genes for PNN components in diseased tissues compared to controls. Using this approach, and contrary to our findings using iPSC-derived MNs, we found that multiple ECM genes frequently associated with PNN components were significantly differentially regulated in human post-mortem ALS tissues across multiple ALS-related mutations (Fig. 3A). This included significant upregulation of *ACAN* and *PTPRZ1* (all cases), *VCAN* and *HAS2* (all except FUS) and *BCAN* (all except FUS and C9orf72), as well as significant down regulation of *HAPLN2* (all cases), *HAPLN3* (all except FUS) and *HAS3* (all except FUS and SOD1). Thus, these data indicate differential expression of genes associated with PNN components in ALS tissue samples, which is not recapitulated in our in vitro model.

MNs are not the only cell type in the CNS that generate PNN components. Indeed, glia such as astrocytes, microglia and oligodendrocytes can also contribute PNN components^10^. Therefore, we again built on an existing meta-analyses of astrocytes formed from hiPSC and mouse lines with ALS mutations^44^. In this analysis, mutations in human (SOD1, C9orf72, FUS) and mouse (SOD1 G93A mutation, *Tardbp* deletion, *Tmem259* deletion) lines were pooled together and corrected for batch effects. We then analysed differential expression of genes associated with PNN components by comparing to healthy controls (Fig. 3B). We found that while mouse ALS astrocytes showed little significant differential regulation of genes for PNN components, for human iPSC lines with ALS mutations, expression of *VCAN*, *TNR*, *PTPRZ1*, and *HAPLN1* was significantly differentially regulated. Although upregulation of *VCAN* reflected changes we had observed in post-mortem tissues (Fig. 3A), differential regulation of the other genes was not consistent. This suggested that although ALS mutations did have some impact on the expression of PNN-associated genes in ALS mutant hiPSC astrocyte cultures, this alone was unlikely to account for the broad regulation of genes for PNN components we observed in human samples at end-stage disease.

In ALS, certain subtypes of MNs are more susceptible to death than others, which can show selective resistance, even at the end stage of disease^6,7^. Indeed, eye movement is often preserved in ALS patients as neurons in cranial nerves III/IV/VI are resistant to cell death^45^. We failed to find evidence of a role for PNNs in ALS using iPSC-derived MNs, but observed significant regulation of ECM genes that are also typical for PNN components in human post-mortem tissues. This led us to hypothesise that our iPSC-derived MNs may not have captured the complexity of the selective resistance/vulnerability of MN populations in vivo. To address this, we next compiled a list of previously published studies that assessed differences in transcriptomic expression between MNs in susceptible and vulnerable areas to ALS using either bulk RNA sequencing or microarray (Table S4). We extracted expression levels from human and mouse data sets collected at various time points and disease states, and from different anatomical sites known to impart resistance or susceptibility in ALS (resistant: Onuf’s nucleus, ON or ocular motor neuron, OMN; vulnerable: spinal motor neuron, SMN). We then plotted logFC differences in genes encoding PNN components or their catabolic regulators, comparing areas that are selectively vulnerable or resistant to MN death as a heat map (Fig. 3C). Many PNN-associated genes were not expressed at detectable levels. Nevertheless, our analysis identified aggrecan (*ACAN*) as significantly downregulated between resistant (OMN) compared to vulnerable (SMN) tissues in certain datasets, including 10-week healthy mouse, 6-week SOD1 mouse, and 9-day iPSC-derived SOD1 ALS mutant MNs. However, it was not significantly differentially expressed in any of the post-mortem human datasets, suggesting a lack of regulation at late-stage disease between vulnerable and resistant areas.

However, when we assessed expression of PNN components in iPSC-derived OMNs and SMNs from SOD1 and C9orf72 ALS mutants, we found that OMNs derived from SOD1 mutant lines had significant differences in expression of *ACAN, HAS2, HAS3, HAPLN1, NCAN, PTPRZ1, HYAL1*, and *HYAL4* relative to SMNs. C9orf72 mutant line-derived OMNs similarly showed significant differences in expression of *BCAN, HAS2, HAPLN1, NCAN, PTPRZ1, MMP9*, and *ADAMSTS4* relative to SMNs. These data may indicate that PNN components are differentially regulated in OMN compared to SMN for C9orf72 and SOD1 ALS mutants. However, we did not observe similar changes in other datasets comparing resistant to vulnerable areas. Therefore, as these MNs were analysed only 9 days after the start of differentiation, and as we similarly observed some differential regulation of PNN components in TDP-43 G298S mutants that did not persist at later time points (Fig. 2D–F), these differences might be attributable to differences in the development of the MNs themselves.

## Discussion

Primary neuron cultures from rodents have been shown to produce PNN-like structures in vitro^33,46,47^. However, to our knowledge, it has not been shown previously that human ESC-/iPSC-derived MNs can also create these ECM structures in vitro. To investigate this, we established co-cultures composed of wild-type human ESC-/iPSC-derived MNs and murine ESC-derived astrocytes. This system robustly generated PNN-like structures that stained positively for aggrecan, tenascin-R, and hyaluronan. iPSC-derived MNs become electrically mature between 2 and 6 weeks of culture^39^—a similar time frame during which we observed deposition of PNN-like structures. These findings mirror those reported in in vivo models where initiation of electrical activity is reported to coincide with PNN formation^48^. Given that PNNs influence neuronal firing patterns^17^ and that hyperexcitability is a hallmark of ALS, our in vitro model may offer a valuable platform to investigate how PNNs—and their loss—affect MN excitability in ALS and other motor diseases.

Following establishment of a culture system in which human ESC-/iPSC-derived MNs form PNN-like structures, we next assessed the impact of ALS-associated mutations and found that the fraction of double-positive MNs (β-III tubulin/PNN component: aggrecan, hyaluronan, tenascin-R) was no different in mutant lines compared to controls. Similarly, qPCR analysis of PNN-associated genes could not detect consistent differences in expression in mutant lines compared to controls, which we confirmed using data provided by a meta-analysis on iPSC-derived MNs formed from lines with additional ALS-associated mutations^43^. These findings may reflect the fact that many core PNN and ECM components are not robustly expressed by MNs themselves, but instead involve contributions from surrounding glial cells. Moreover, many of the molecular constituents of PNNs are not unique to these structures, but are shared with the broader ECM, which is known to be extensively remodelled in ALS spinal cord tissue^23,49^. ALS mutant astrocytes show increased expression of ECM-related genes and reduced expression of genes associated with synaptic support^44^. In this study, we deliberately adopted a reductive system in which ALS-associated mutations were confined to MNs, enabling assessment of MN-intrinsic contributions to PNN formation. However, our study design does not definitively resolve the cellular source of PNN components and so does not exclude potential non-cell-autonomous contributions from surrounding cell types. Future studies incorporating genotype-matched neuron-glia co-cultures will be important to dissect how non-cell-autonomous interactions may shape synaptic activity and contribute to PNN and ECM dysregulation in ALS^44,50^.

Using the interactive analysis tool provided by Ziff et al.^43^ to interrogate post-mortem ALS transcriptomic datasets, we identified significant alterations in multiple ECM components associated with PNNs, including both up- and downregulated constituents. This mixed pattern likely reflects complex compensatory or uncoordinated responses arising from multiple cell types within degenerating tissue. Post-mortem samples represent end-stage disease, when many MNs have already been lost, and the molecular processes driving disease initiation and progression may no longer be active. In this context, it is possible that iPSC-derived MNs model earlier phases of disease or capture only a subset of ALS-relevant mechanisms. Indeed, transcriptional changes detected in bulk tissue should be interpreted cautiously, as they may reflect tissue degeneration, shifts in cellular composition, or secondary responses rather than primary alterations in PNN assembly by MNs.

Recent work has highlighted a broader role for ECM remodelling in ALS beyond PNNs. In particular, Milioto et al. identified a neuron-intrinsic ECM gene signature induced in C9orf72 ALS/FTD models in response to poly-GR and poly-PR toxicity, which was proposed to act as a neuroprotective stress response^51^. This programme encompassed a wide range of ECM components and was observed in vivo under conditions of pronounced proteotoxic stress. Our findings do not contradict this work, but instead suggest that while neurons can mount adaptive ECM responses under stress, the formation of PNN-like structures is not intrinsically altered by ALS-associated mutations. Instead, they support a model in which ECM changes in ALS are context-dependent and may require disease-relevant stressors or multicellular interactions not captured in simplified MN cultures.

Kaplan and colleagues showed that MMP9 is selectively expressed by vulnerable MNs in murine models of ALS, and that genetic deletion of MMP9 preserves neuromuscular junction innervation, improves motor performance, and extends survival^28^. To explore whether similar PNN-related mechanisms distinguish resistant and vulnerable MN populations, we performed a meta-analysis of publicly available datasets comparing resistant and susceptible MNs in ALS. However, we could not find convincing evidence implicating PNNs in the resistance of ocular (or Onuf’s nucleus) MNs, and many PNN-related transcripts were not detected. Thus, our analyses do not support MN-intrinsic differences in PNN-related gene expression between resistant and vulnerable populations, pointing instead to roles for additional cellular or contextual factors.

PNNs are assembled and degraded by multiple CNS cell types, with glial cells playing central roles in their formation, integrity, and remodelling. Recent reports have identified microglia that express MMP9 surrounding denuded MNs, and have even shown that microglia can engulf PNNs^21^. Moreover, a single-nucleus RNA sequencing study of ALS patient tissue has revealed elevated microglial expression of *SQSTM1/p62*^50^, a known promoter of ECM degradation^52^. Consistent with a protective role for PNNs, α-MNs lacking PNNs exhibit increased levels of the protein oxidation marker 3-nitrotyrosine, linking PNN loss to oxidative stress^21^. Together, these observations cautiously support a model in which microglial-mediated PNN degradation may weaken local neuronal protection and contribute to MN vulnerability in ALS.

We observed a modest but significant shift in TDP-43 subcellular localisation in ALS mutant MNs, indicating altered TDP-43 regulation without overt nuclear depletion. Prior studies have shown that pronounced nuclear loss of TDP-43, as observed in severe in vivo models or acute depletion paradigms, leads to widespread molecular dysregulation and is associated with disease contexts in which genes linked to MN vulnerability, including MMP9, are altered^53–56^. Consistent with this distinction, we did not detect changes in MMP9 expression in our mutant lines, despite MMP9 being a recognised marker of selectively vulnerable MN populations in ALS models^28^. These findings suggest that the degree of TDP-43 perturbation present in our system falls below the threshold required to induce MMP9 dysregulation, which may instead require prolonged nuclear loss, additional cellular stressors, or non-cell-autonomous influences. Examination of published datasets describing widespread splicing defects following TDP-43 depletion did not reveal consistent misregulation of core PNN components, including aggrecan, versican, brevican, HAPLN family members, or tenascin-R^53–56^, indicating that PNN-associated genes are not primary or early targets of TDP-43 loss-of-function. Thus, while our data do not support an MN-intrinsic effect of ALS-associated mutations on PNN gene expression or deposition, this does not preclude a role for PNNs in shaping selective vulnerability through other mechanisms. PNNs regulate synaptic stability, excitability, and perisynaptic organisation^14,17^, processes that involve molecular pathways known to be sensitive to TDP-43 dysfunction. In this context, PNNs may influence ALS pathology indirectly by modulating the synaptic and structural environment in which TDP-43-dependent processes operate, rather than through direct transcriptional dysregulation of PNN components within MNs themselves.

Recent cell-type-resolved transcriptomic studies provide additional context for interpreting ECM and PNN alterations in ALS^50,57,58^. For example, data from O’Neill et al. show that regulation of PNN-associated components, including proteoglycans, link proteins, and matrix-remodelling enzymes, is differentially regulated within glial-associated disease contexts, whereas a coordinated PNN-related transcriptional programme is not evident within neuronal populations^57^. When considered alongside our finding that ALS-associated mutations in human MNs do not intrinsically impair PNN-like structure formation in vitro, these observations suggest that PNN-related signatures detected in ALS tissues are likely not explained by neuronal mechanisms alone, but may instead reflect contributions from additional cellular sources and context-dependent ECM remodelling. This distinction underscores the need for future experimental systems that incorporate glial cells and more physiologically relevant tissue architectures to understand how ECM remodelling influences PNN organisation and MN vulnerability in ALS.

Together, our findings demonstrate that human iPSC-derived MNs can form PNN-like structures in vitro, providing a cell culture model to study their function and regulation in ALS and other motor diseases. While our data suggest that MN-intrinsic mechanisms are not sufficient to recapitulate PNN-related alterations described in ALS animal models, changes in PNN-associated gene expression observed in post-mortem tissues are consistent with contributions from non-neuronal cells in modulating these neuroprotective structures. This model lays the groundwork for future mechanistic studies into how cell-type interactions and matrix remodelling contribute to MN vulnerability in ALS.

## Materials and methods

### iPSC culture

Human induced pluripotent stem cells (iPSC) or human embryonic stem cells (ESC) (Table 1) were cultured in StemMacs™ iPSC brew supplemented with penicillin-streptomycin (1X) on laminin-521 basement matrix (LN521, Thermo Fisher, #A29249) diluted 1:50 (0.4 µg/cm^2^) in phosphate-buffered saline (PBS) containing calcium and magnesium (Thermo Fisher, #14040117). iPSCs were passaged as single cells when ~70% confluent. For passaging, confluent cells were detached from the culture plate by incubating with TrypLE express (Invitrogen) for 4 min at 37 °C. iPSCs were then diluted in Dulbecco’s modified Eagle’s medium (DMEM) 1:10 (Thermo Fisher, #12491015) and centrifuged at 260 × *g* for 4 min before plating the required number of cells in iPSC media supplemented with 10 µm Y-27632 (Tocris, Cat#1254) for 24 h. iPSC were maintained at 37°C with 5% CO_2_/95% air. Media was replenished every 24–48 h.

**Table 1:** List of cell lines and their sources

Cell line	Details of source
H9 human ESC line (Wildtype, WT)	WiCell
M211R2 C9orf72-mutant human iPSC line (C9-3)	Mehta et al. *Acta Neuropathol* 2021^60^
DN1qV4 C9orf72-mutant human iPSC line (C9-2)	Mehta et al. *Acta Neuropathol* 2021^60^
Isogenic DN1qV4 control human iPSC line (Iso-C9-2)	Mehta et al. *Acta Neuropathol* 2021^60^
TDP-43 G298S-mutant human iPSC line (TDP-43-G298S)	Barton et al. *Brain Commun* 2021^61^
Isogenic TDP-43 G298S control human iPSC line (Iso-TDP-43-G298S)	Harley et al. *Cell Rep* 2023^39^
H9 human ESC line, Wildtype, WT, Hb9::CD14 transgenic	Harley et al. *Cell Rep* 2023^39^
M211R2 C9orf72-mutant human iPSC line C9-3, Hb9::CD14 transgenic	Harley et al. *Cell Rep* 2023^39^
DN1qV4 C9orf72-mutant human iPSC line, C9-2, Hb9::CD14 transgenic	This study
Isogenic DN1qV4 control human iPSC line, Iso-C9-2, Hb9::CD14 transgenic	This study
TDP-43 G298S-mutant human iPSC line, TDP-43-G298S, Hb9::CD14 transgenic	Harley et al. *Cell Rep* 2023^39^
Isogenic TDP-43 G298S control human iPSC line, Iso-TDP-43-G298S, Hb9:CD14 transgenic	Harley et al. *Cell Rep* 2023^39^
H9 human ESC line, Wildtype, Hb9::CD14/CAG::CAG::ChR2-YFP transgenic	Harley et al. *Cell Rep* 2023^39^
M211R2 C9orf72-mutant human iPSC line C9-3, Hb9::CD14/CAG::CAG::ChR2-YFP transgenic	Harley et al. *Cell Rep* 2023^39^
TDP-43 G298S-mutant human iPSC line TDP-43-G298S, Hb9::CD14/CAG::CAG::ChR2-YFP transgenic	Harley et al. *Cell Rep* 2023^39^
Isogenic TDP-43 G298S control human iPSC line Iso-TDP-43-G298S, Hb9::CD14/CAG::CAG::ChR2-YFP transgenic	Harley et al. *Cell Rep* 2023^39^
IB10 mouse embryonic stem cell (mESC) line, GFAP::CD14/CAG:: GDNF transgenic	Bryson et al., Science 2014^38^

### Engineering of iPSC lines

To enable downstream purification of MNs and astrocytes, human iPSCs and mESCs were engineered to allow for cell type-specific magnetic activated cell sorting (MACS). The mESC line had been engineered previously with GFAP::CD14^38^. For MN differentiation, wildtype, TDP-43-G298S and Isogenic TDP-43-G298S human iPSC lines had all been previously engineered with Hb9::CD14^39^. To create C9orf72 mutant lines and their isogenic controls (C9-3, C9-2, iso-C9-2), we inserted the Hb9::CD14 (Addgene, #204344) construct via electroporation. 1 µg/µl plasmid DNA was added to OptiMEM Reduced-Serum Medium (Gibco, 11058021). iPSCs were detached from LN521-coated plates using TrypLE Express, washed once with DMEM, and then twice with Opti-MEM Reduced Serum Medium™ before concentrating cells to 1 million iPSC/90 µl. For Hb9::CD14 insertion into the AAVS1 (Adeno-associated virus integration site 1) locus, 5 µg Hb9::CD14 plasmid, 2.5 µg TALEN-Left (Addgene #52341) and 2.5 µg TALEN-Right (Addgene #52342) were mixed with iPSC. Each iPSC/plasmid mixture was added to 2 mm electroporation cuvettes (GeneFlow, E6-0062) and electroporated. Post-electroporation, iPSCs were collected and replated in LN521-coated 60 mm dishes in pre-warmed iPSC media supplemented with 10 µm Y-27632 for the first 24 h, and were allowed to reach 70% confluency. iPSCs were then treated with 0.5 µg/µl puromycin for 5 days for the selection of transfected cells. After recovery, single iPSCs were plated by limiting dilution into LN521-coated 96-well plates (0.5 cells/well). Surviving iPSC colonies were dissociated using TrypLE, and genomic DNA was isolated using QuickExtract (Cambio/Epicentre, #QE09050^59^). Genomic DNA was amplified by PCR and screened for homozygous insertion of the Hb9::CD14 plasmid.

### Generation of motor neurons (MNs) from iPSC lines

MNs were generated from iPSC lines as described previously^37^. Single iPSCs were plated in suspension culture plates in MN differentiation media (N.MND, Table 2) supplemented with 10 μM Y-27632, 20 μM SB431542, 0.1 μM LDB193189, and 3 μM CHIR. On day 2, media was replaced with N.MND media supplemented as above with the addition of 100 nM RA and 500 nM SAG. On day 4, media was replaced with N.MND media supplemented with 20 μM SB431542, 0.1 μM LDB193189, 100 nM RA and 500 nM SAG. On day 7, the media was changed to N.MND media supplemented with 100 nM RA and 500 nM SAG. On day 9, the media was changed to N.MND media supplemented with 500 μM ascorbic acid, 0.1 μM compound E, 100 nM RA and 500 nM SAG. On day 11, media was changed to N.MND media supplemented with 500 μM ascorbic acid, 0.1 μM compound E, 100 nM RA, 10 ng/mL BDNF, 10 ng/mL GDNF, 10 ng/mL CNTF, 10 ng/mL NT3, 10 ng/mL IGF1, 10 μM forskolin, 100 μM IBMX and 500 nM SAG. On day 14, MNs were purified using MACS.

**Table 2:** Composition of N.MND

Component	Volume in 50 mL	Manufacturer
Advanced DMEM/F12	24 mL	Thermo Fisher Scientific
Neurobasal media	24 mL	Thermo Fisher Scientific
Penicillin-streptomycin, 100X	0.5 mL	Thermo Fisher Scientific
L-Glutamine (200 mM)	0.5 mL	Thermo Fisher Scientific
NeuroBrew-21 supplement without vitamin A (50X)	0.5 mL	Miltenyi Biotech
N-2supplement	0.25 mL	Thermo Fisher Scientific
β-mercaptoethanol1000x	200 µL	Thermo Fisher Scientific

### Generation of astrocytes from mESC

The mouse ESC E14-IB10 subclone G6 was previously engineered to express GFAP::CD14/CAG::GDNF^40^. mESC were cultured in mESC maintenance media (Table 3) on LN521-coated plates (0.08 μg/cm^2^) until 70% confluent, with maintenance media replaced every 1–2 days. 1 million mESC were placed in 10 cm^2^ suspension plates with astrocyte media (Table 4) for 2 days. On day 2, EBs were collected, split 1:4 and then cultured in astrocyte media supplemented with 0.5 μM SAG and 1 μM RA for 3 days. On day 5, EBs were collected and replated in astrocyte media in T175 flasks coated with Matrigel (1:100 diluted in DMEM) and cultured in astrocyte media for a further 7 days until purification by MACS.

**Table 3:** Composition mESC maintenance medium

Component	Volume in 50 mL	Manufacturer
DMEM/F12 Medium (1X)	22.5 mL	Thermo Fisher Scientific
Neurobasal media	22.5 mL	Thermo Fisher Scientific
Penicillin-streptomycin, 100X	0.5 mL	Thermo Fisher Scientific
L-Glutamine (200 mM)	0.5 mL	Thermo Fisher Scientific
MACS NeuroBrew-21 without vit A (50X)	0.5 mL	Miltenyi Biotech
N-2 supplement	0.25 mL	Thermo Fisher Scientific
β-mercaptoethanol1000x	50 µL	Thermo Fisher Scientific
PD0325901	5 µL	Tocris
CHIR99021	15 µL	Tocris
Plasmocin Prophylactic (25 mg/ml)	10 µL	InvivoGen
2 inhibitor/leukemia inhibitory factor (2iLIF) supernatant (COS7/pCAGGS::LIF)	0.5 mL	
ESC-qualified Fetal BSA	1 mL	Gibco
5% Sterile BSA fraction V	1 mL	Roche

**Table 4:** Composition of astrocyte medium

Component	Volume in 50 mL	Manufacturer
Advanced DMEM/F12	24 mL	Thermo Fisher Scientific
Neurobasal media	24 mL	Thermo Fisher Scientific
Penicillin-streptomycin, 100X	0.5 mL	Thermo Fisher Scientific
L-Glutamine (200 mM)	0.5 mL	Thermo Fisher Scientific
NeuroBrew-21 supplement without vitamin A (50X)	0.5 mL	Miltenyi Biotech
N-2supplement	0.25 mL	Thermo Fisher Scientific
β-mercaptoethanol1000x	200 µL	Thermo Fisher Scientific
5% Sterile BSA fraction V	1 mL	Roche

### Magnetic activated cell sorting (MACS) purification of MNs and astrocytes

MACS purification of MNs was performed as described previously^39^. Briefly, day 14 EBs were dissociated using trypsin-EDTA supplemented with 10 U/mL DNase-I (Merck, #4536282001) for 8–10 min. DMEM supplemented with 10 U/mL DNase-I and 10% FBS (Thermo Fisher, #26140079) was then added to the EB/trypsin-EDTA mixture and centrifuged at 500 rpm for 4 min and the supernatant was aspirated. To obtain single cells, EBs were resuspended in 500 µL DMEM + DNase-I (10 U/mL) and were mechanically disrupted by pipetting up and down 10-12 times. 1 mL DMEM + DNase-I (10 U/mL) was then added, and the remaining EBs were allowed to settle for 5 min. 1 mL of supernatant was removed and placed in a new collection tube, and this process was repeated 3 times. EB supernatant solution was then passed through a 40 µm strainer, centrifuged and washed once in MACS buffer (90% PBS, 10% 0.5% BSA and 10 U/mL DNase-I). 3 µg/mL anti-CD14 diluted in MACS buffer was added to the cell suspension and incubated on a rotator for 15 min at 4 °C. Cells were washed once with MACS buffer before adding anti-mouse IgG microbeads (1:10, Miltenyi, #130-048-402) and the solution was again incubated on a rotator for 20 min at 4 °C. Following an additional wash in MACS buffer, cells were resuspended in MACS buffer before passing through a MACS column (Miltenyi, #130-042-201) that had been pre-washed with MACS buffer. Cells retained on the column were then resuspended in N.MND media. To purify mESC-derived astrocytes, a similar procedure was used, with the exception that, as astrocyte cultures were adherent and grown in T175 flasks, trypsin-EDTA supplemented with 10 U/mL DNase-I (15 min) was used to detach cells before being passed through a 40 µm strainer. Eluted cells were resuspended in astrocyte media.

### Establishment of iPSC-derived MNs and mESC-derived astrocyte co-cultures

Purified populations of MNs and astrocytes were used to generate co-cultures. Astrocytes were plated in astrocyte media 5-7 days before MNs were added to allow recovery and cell spreading, before plating MNs on top. For co-cultures, a 50:50 mixture of astrocyte medium:N. MND media was used with 10 µm Y-27632 added for the first 24 h of culture.

### qPCR analysis of MNs-astrocyte co-cultures

Total RNA was isolated from cells using an RNeasy Mini Kit (Qiagen, 74104), following the manufacturer’s guidelines, and RNA quantified using a NanoDrop 2000 (Thermo Fisher Scientific). Single-stranded cDNA was generated using a High-Capacity cDNA Reverse Transcription Kit (Thermo Fisher Scientific, #4368814) using the manufacturer’s guidelines. 6.25 ng/µL cDNA in a final volume of 10 µL was used for qPCR. A mixture of 50% Fast SYBR Green PCR Master Mix (Thermo Fischer Scientific, 4385612), 35% water (Thermo Fisher Scientific, #AM9935) and oligonucleotide primers (2.5% forward and 2.5% reverse primer) (Sigma-Aldrich, Table 5) was prepared for each primer pair. This mixture was added to each well with 1 µL cDNA solution and qPCR performed on a CFX384 Touch Real-Time PCR Detection System (BioRad) at the following settings: 95 °C 10 min, 40 cycles of 95 °C for 30 s and 60 °C for 1 min, 75 °C for 10 s, 95 °C for 10 s. Housekeeping genes and data was analysed either by -∆Ct (threshold cycle value) or Comparative CT Method (2∆Ct) using *ACTB* and *GAPDH* as housekeeping genes. Efficiency tests were performed on primers using human spinal cord total RNA (Takara bio, #636554), which confirmed that all had efficiencies between 85 and 105%.

**Table 5:** Primers used for qPCR

Target gene	Forward Primer (5’-3’)	Reverse Primer (5’-3’)
*ACAN*	CCCCTGCTAT-TTCATCGACCC	GACACACGGC-TCCACTTGAT
*ACTB*	CACCATTGGCA-ATGAGCGGTTC	AGGTCTTTGCG-GATGTCCACGT
*ADAMSTS4*	CTGGCACCTA-CCTGACTGG	GTAACACGCC-TAACAGGGCT
*BCAN*	TGGAAGGAGA-CAGCTCAGAGG	CGGGACAGGA-AAGTCCACTTG
*CHAT*	CAGCCCTGCC-GTGATCTTT	TGTAGCTGAGT- ACACCAGAGATG
*GAPDH*	GTCTCCTCTGA-CTTCAACAGCG	ACCACCCTGTT-GCTGTAGCCAA
*HABP4*	GAAGCGGACTC-CTAGAAGAGG	CATAGGGTCGG-TATTCCCTGTA
*HAPLN1*	TCTGGTGCTGA-TTTCAATCTGC	TGCTTGGATGT-GAATAGCTCTG
*HAPLN2*	TTTCGGGGC-TAGACCAGTG	AATTCTCGGCG-TAGCAGTAGG
*HAPLN3*	TCCTACGGAC-TGCCCTTCTAC	AGCTTCACTCCA-TTAAGGAGGT
*HAPLN4*	AACGTCACGC-TGCAAGACTAC	GTCAGCTTGTA-TCGGCCTCC
*HAS2*	CTCTTTTGGAC-TGTATGGTGCC	AGGGTAGGTTA-GCCTTTTCACA
*HAS3*	CGCAGCAACT-TCCATGAGG	AGTCGCACACC-TGGATGTAGT
*HYAL1*	TATGGCCCA-AGGCTTTAGGG	GCTACCACATC-GAAGACACTGA
*HYAL3*	TGCCTCTTCTT-TTCCCTGCTG	GGGAGGGTTG-ACTGTAAACTG
*HYAL2*	GAGCACTACA-TTCGGACACAG	GATAACCGGCG-ATACACATCTT
*MMP9*	AGACCTGGGC-AGATTCCAAAC	CGGCAAGTCT-TCCGAGTAGT
*NCAN*	GCCTTTGCCT-CCCCAGCTAT	CTGGAACTT-ATGCCAGCCGC
*PTPRZ1*	GCCTGGATT-GGGCTAATGGAT	CAGTGCTCCTG-TATAGGACCA
*SEMA3A*	GTGCCAAGGC-TGAAATTATCCT	CCCACTTGCAT-TCATCTCTTCT
*TBP*	TGTATCCACAG-TGAATCTTGGTTG	GGTTCGTGGCTC-TCTTATCCTC
*TNC*	TCCCAGTGTT-CGGTGGATCT	TTGATGCGAT-GTGTGAAGACA
*TNR*	GGGGAGATG-TGCCAACGGTA	CAGCCACTCG-CAAGTCCTCT
*VCAN*	TGATGCGGG-TCTTTACCGCT	GGATGACCAATT-ACACTCAAATCAC

### Immunocytochemistry staining of co-cultures

Half the volume of media was removed from wells and replaced with 4% w/v paraformaldehyde (PFA) (Thermo Fisher Scientific, 11586711) supplemented with 15% sucrose. After 10 min, half of this solution was removed, and another fresh volume of PFA/sucrose was added to the well for another 10 min. The total volume was then removed, and the well was washed twice with PBS. A combination permeabilisation-blocking step was then performed with 3% v/v Horse Serum (Sigma-Aldrich, #H0146) in PBS-T (0.1% Triton X-100 (Thermo Fisher Scientific) in PBS) for 30 min. Cultures were incubated overnight at 4 °C with primary antibodies diluted as indicated in Table 6. The following day, wells were washed three times with DPBS before adding secondary antibodies. Secondary antibody incubation occurred at room temperature for 45 min before washing 3 times with DPBS. Samples were then imaged using either a Leica TCS SP8 (Leica Microsystems) or Zeiss LSM 980.

**Table 6:** Antibodies used for immunostaining

Antibody	Catalogue number	Species	Supplier	Concentration
Aggrecan core protein	bs-1223R-BSS	Rabbit	Bioss	1:200
Hyaluronic acid binding protein	385911	Biotinylated	Sigma-Aldrich	1:200
hTenascin R pAb	AF3865-SP	Goat	RnD Systems	1:200
Wisteria floribunda agglutinin	L1516	Biotinylated	Sigma-Aldrich	1:200
Beta-III tubulin	MAB1195	Mouse	RnD Systems	1:1000
Glial acidic fibrillary protein	2.2B10	Rat	Thermo Fisher	1:250
TDP-43	107-82-2-AP	Rabbit	Protein Tech	1:300
Streptavidin AlexaFluor™ 647	S32357		Abcam	1:1000
Donkey anti-Mouse IgG, AlexaFluor™ 488	A-21202	Donkey	Thermo Fisher	1:1000
Donkey anti-Mouse IgG2a, AlexaFluor™ 647	A-21241	Goat	Thermo Fisher	1:1000
Donkey anti- Rabbit IgG, AlexaFluor™ 568	A10042	Donkey	Thermo Fisher	1:1000
Donkey anti-Goat IgG, AlexaFluor™ 568	A-11057	Donkey	Thermo Fisher	1:1000
Donkey anti-Rat IgG, AlexaFluor™ 568	A78946	Donkey	Thermo Fisher	1:1000
Donkey anti-Rat IgG, AlexaFluor™ 647	A48272	Donkey	Thermo Fisher	1:1000

### Statistics and reproducibility

Unless otherwise stated, data are presented as mean ± standard error of the mean (SEM). Details of each test used are given in the relevant figure legends. Data were analysed using Graphpad Prism, Version 10 (Graphpad Software, San Diego, California, USA).

### Reporting summary

Further information on research design is available in the Nature Portfolio Reporting Summary linked to this article.

## Supplementary information

Supplementary Figs. and Tables

Supplementary Data 1

Description of Additional Supplementary Files

Reporting summary

## Data Availability

Cell lines are available on request and through appropriate MTAs. Data are either publicly available, available within the file Supplementary Data 1, or are available upon reasonable request to the corresponding author.

## Code availability

Code are available within the supplementary information included with this manuscript.
