# Supplementary material for: ALS mutations do not alter perineuronal net formation in human stem cell-derived motor neurons
Source: Commun Biol. 2026 May 19;9:1062. doi: 10.1038/s42003-026-10244-6 (PMC13454605; doi:10.1038/s42003-026-10244-6)
Supplement: Supplementary file 1 [file 42003_2026_10244_MOESM1_ESM.pdf]

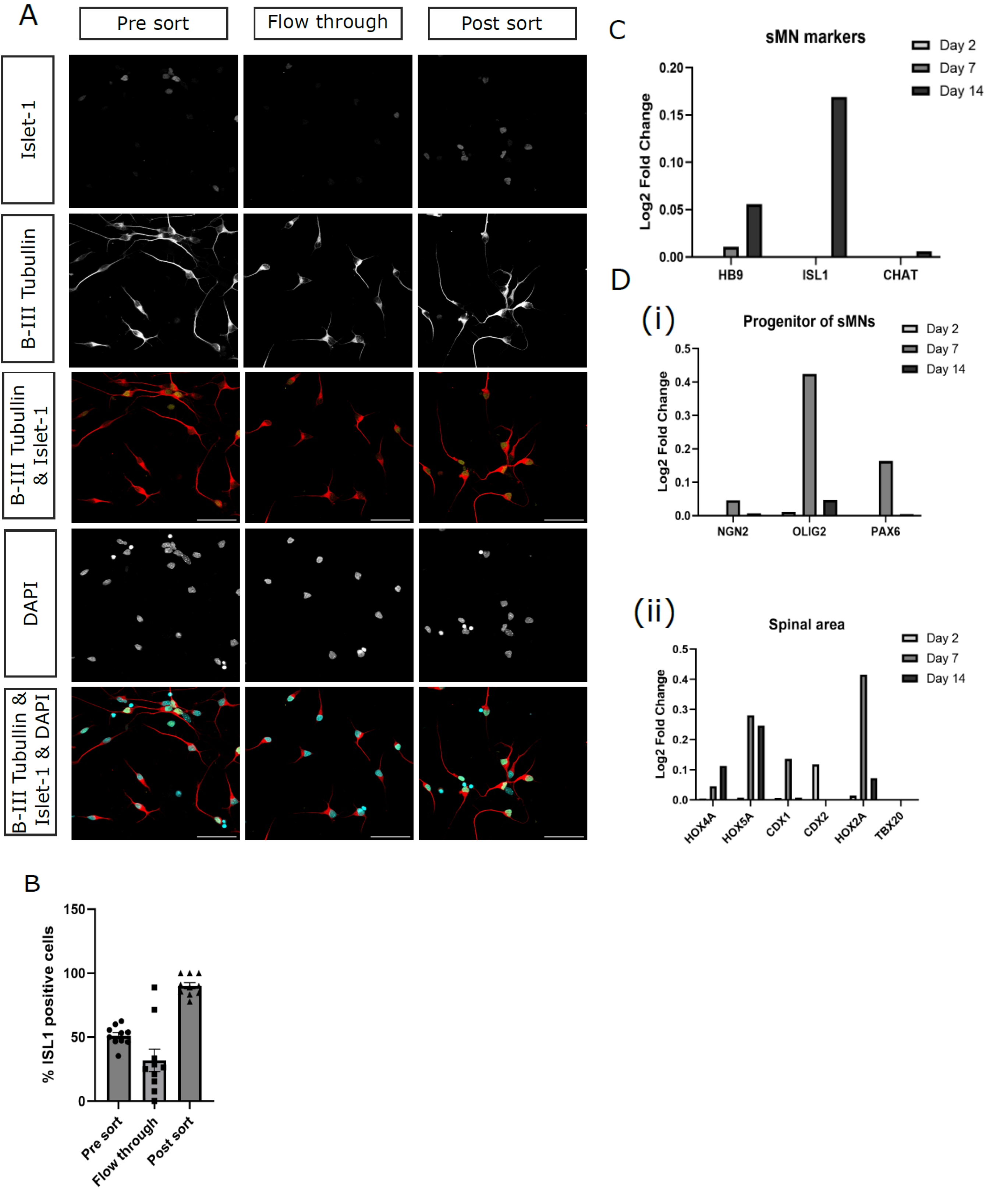

Figure S1: Quantification of MACS efficiency at retaining MNs. A) Representative immunofluorescence images of cells from pre-sort, flow-through, and post-sort fractions following MACS performed on day 14 embryoid bodies (Ebs) derived from hiPSC and stained for β-III tubulin, Islet-1 and DAPI. B) Quantification of islet positive MNs obtained from pre-sort, flow-through, and post-sort fractions following MACS of day 14 EBs. Five images were analysed per fraction; N = 2 independent differentiations. C) qPCR analysis of MN marker expression (*ISL1, CHAT, HB9*) in RNA extracted from wild-type EBs harvested at days 2, 7, and 14 of differentiation. D) qPCR analysis of spinal MN progenitor and patterning markers in wild-type EBs harvested at days 2, 7, and 14, including (i) *NGN2, OLIG2*, and *PAX6*, and (ii) *HOXA4, HOXA5, CDX1, CDX2, HOXA2,* and *TBX20*. For all qPCR analyses, N = 3 independent differentiations.

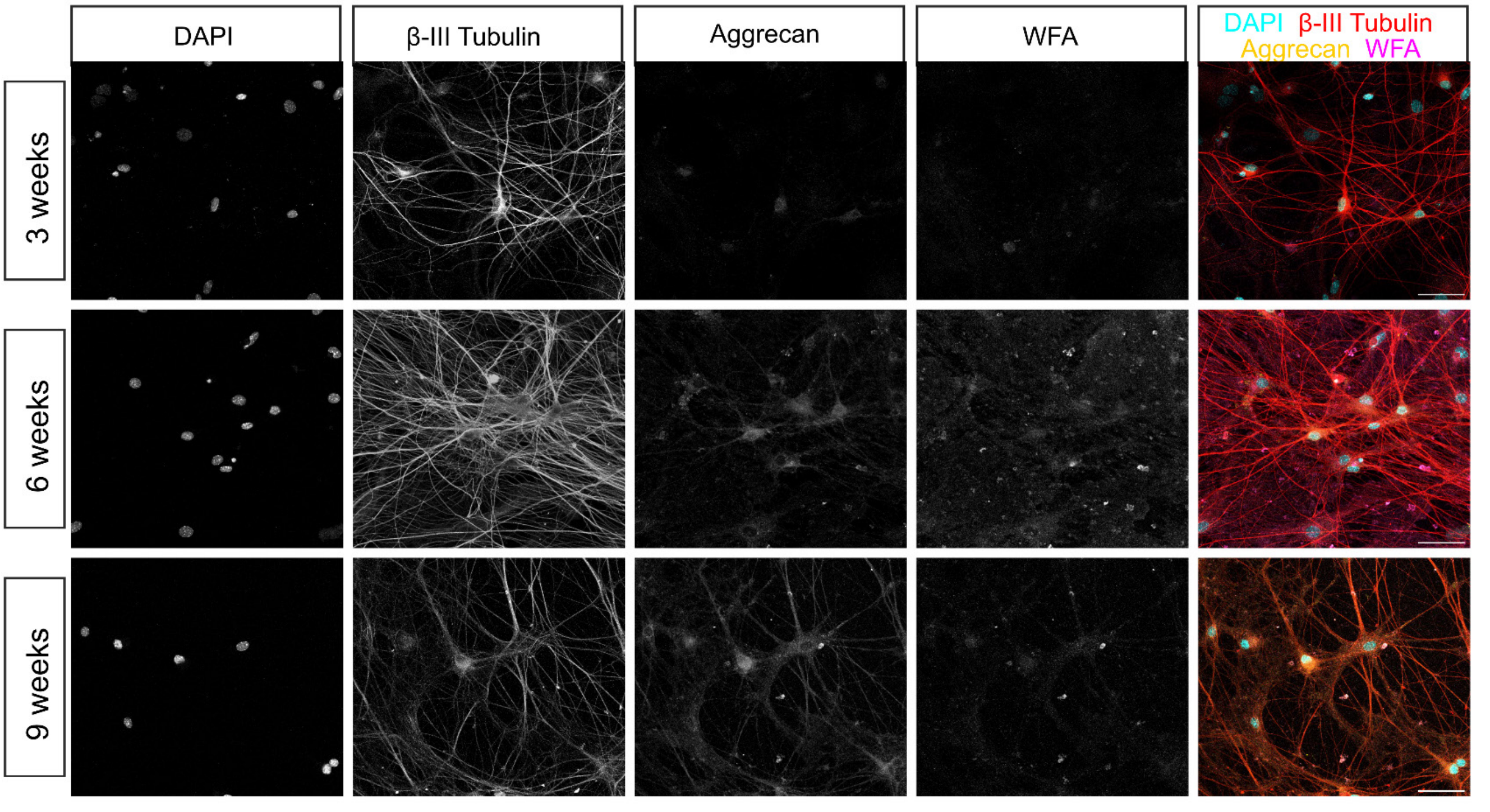

Figure S2: Representative confocal microscopy images of 3-, 6- and 9-week co-cultures of human ESC-derived MNs and astrocytes. Staining for β-III tubulin, aggrecan, Wisteria Floribunda Agglutin (WFA) and DAPI. WFA staining highlights similar structures to antibodies against aggrecan. Scale bar = 25µm. N=2.

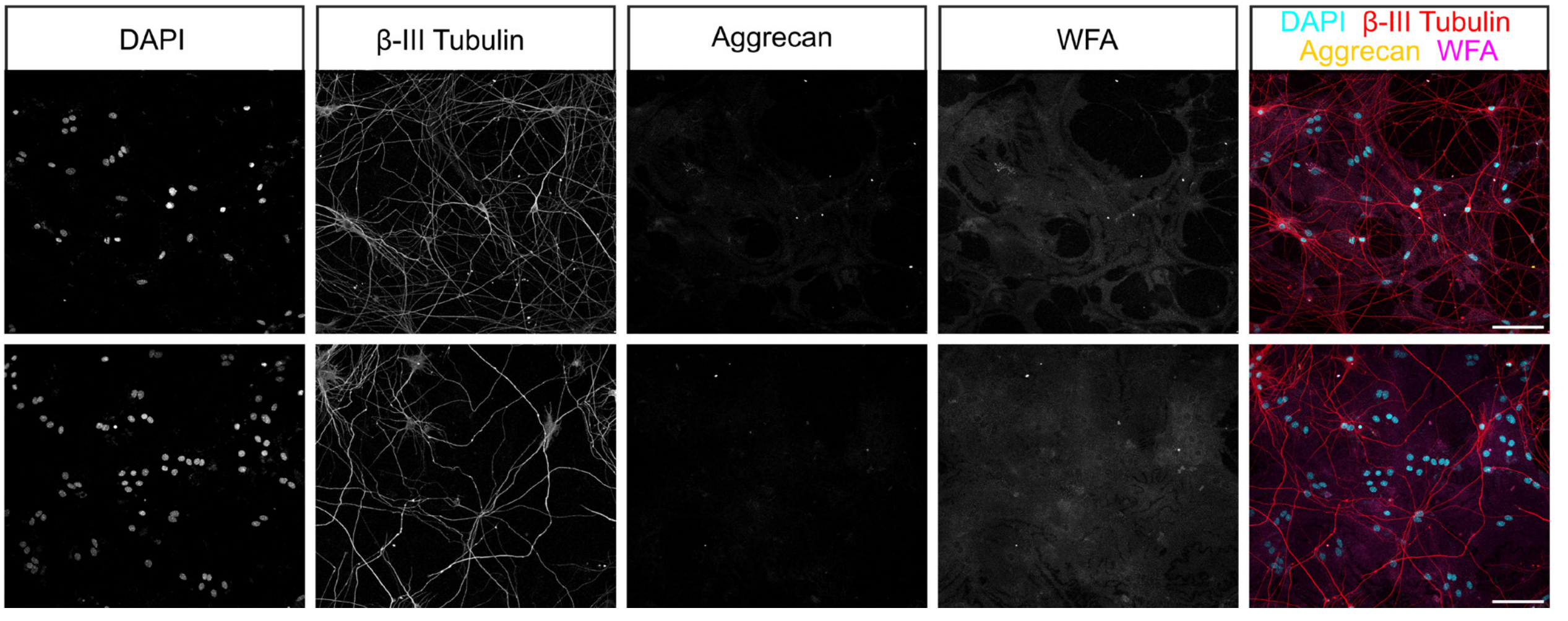

Figure S3: Representative confocal microscopy images of 2-week co-cultures of WT human ESC-derived MNs and astrocytes stained for β-III tubulin, aggrecan, Wisteria Floribunda Agglutin (WFA) and DAPI. PNN-like structures are not apparent at this stage. Scale bar = 100µm. N=2.

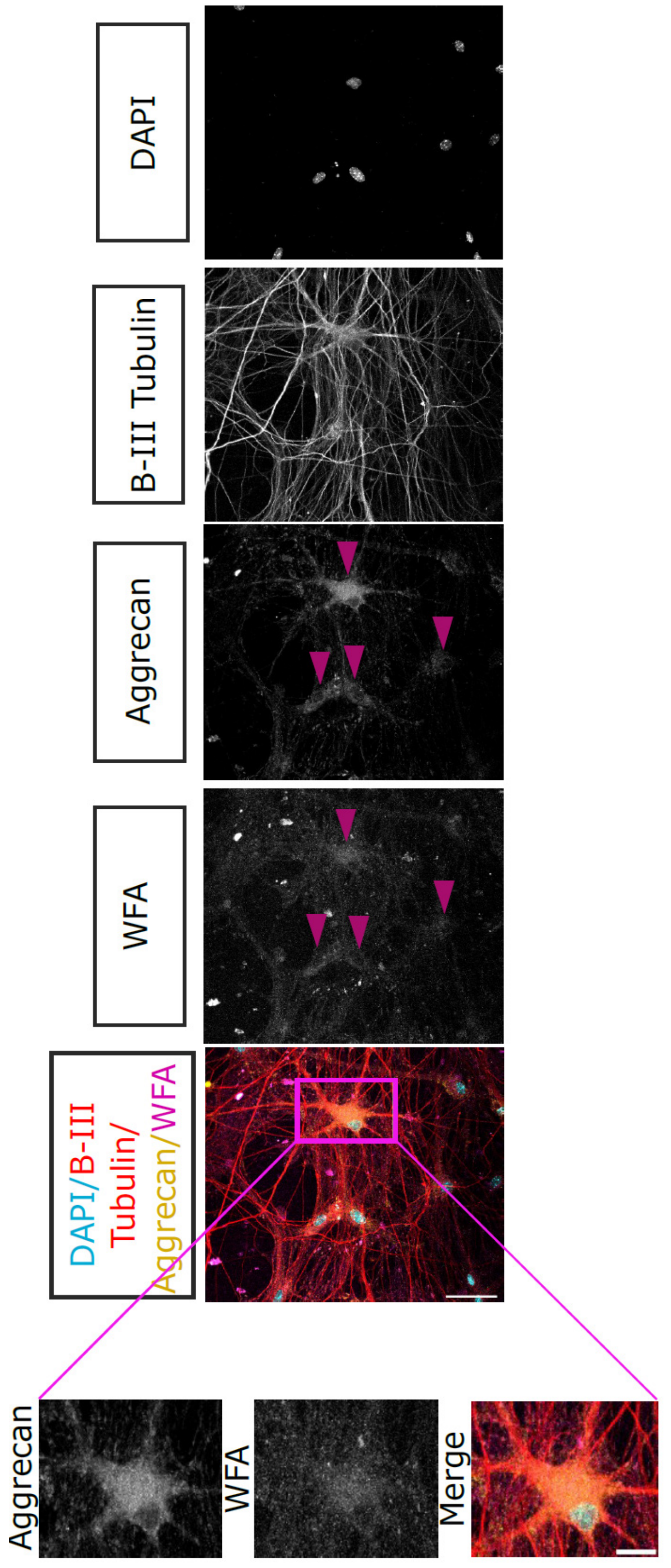

Figure S4: Representative confocal microscopy images of 9-week co-cultures of WT human ESC-derived MNs and astrocytes stained for β-III tubulin, aggrecan, Wisteria Floribunda Agglutin (WFA) and DAPI. Pink arrowheads point towards areas corresponding to β-III tubulin positive MNs. Scale bar = 50µm. Images acquired on Leica SP8 scanning laser confocal microscope. N=2.

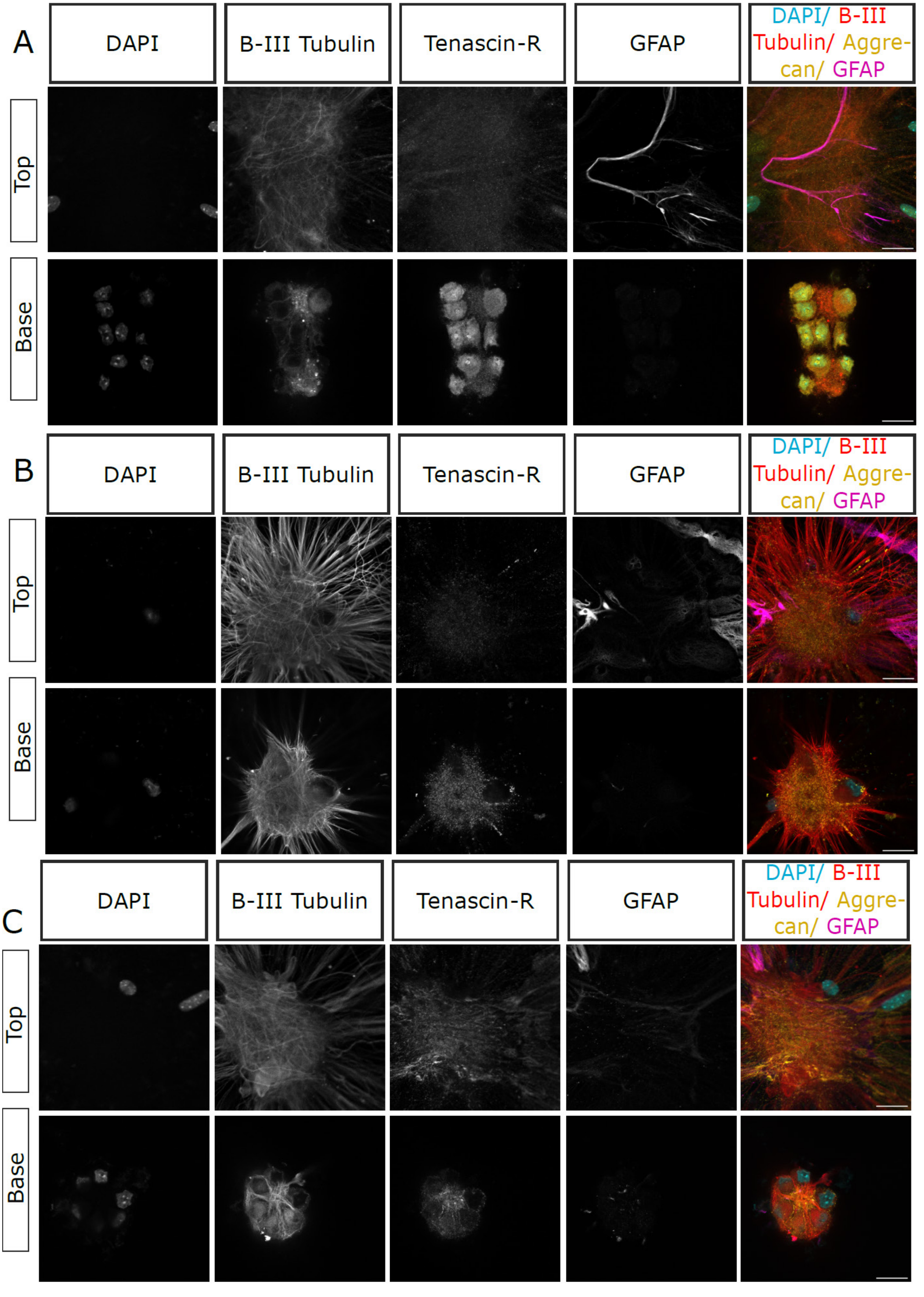

Figure S5: PNN components do not accumulate around astrocytes. Representative confocal microscopy images captured after 9 weeks of culture showing staining for PNN components A) aggrecan, B) hyaluronan, and C) tenascin-R captured from both the base and top of the cultures. Staining for PNN components almost never co-localised with GFAP+ astrocytes (GFAP+) which were present at the base of co-cultures. PNN instead accumulated around β-tubulin+ MNs. Scale bar = 25µm. N=3.

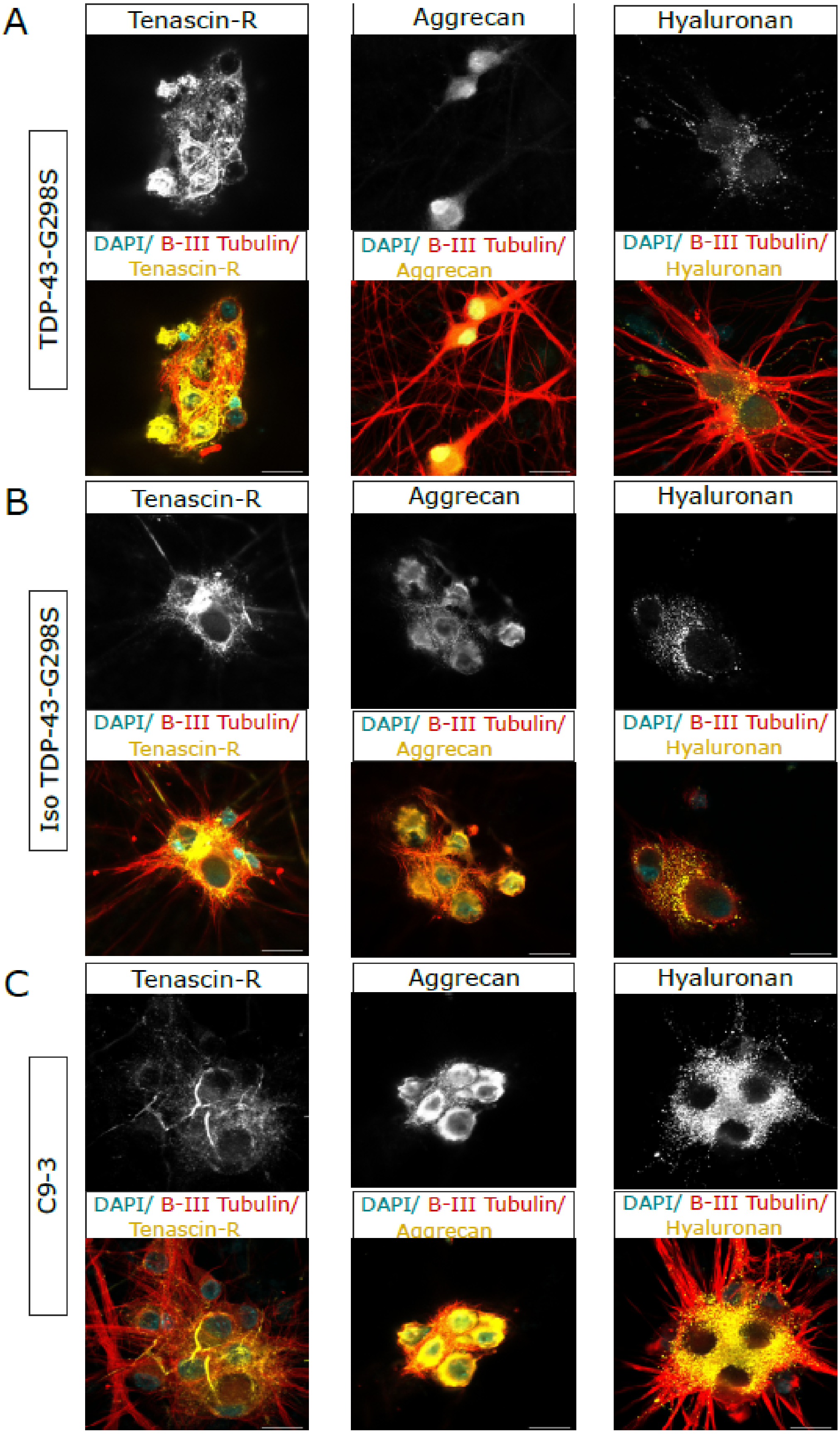

Figure S6: Representative confocal microscopy images of co-cultures stained for β-III tubulin, aggrecan, hyaluronan and tenascin-R after 9 weeks in culture. Scale bar = 25μm. Zeiss LSM confocal microscope, 63X. Representative images from 2 healthy lines (H9 and Iso-TDB-43-G298S) and two ALS line (C9-3 and TDB-43-G298S) all produce PNN components robustly. N=3.

Hyaluronan				
Tukey's multiple comparisons test	Mean Diff.	Below threshold?	Summary	Adjusted P Value
3wks vs. 6wks	-32.35	Yes	**	0.0047
3wks vs. 9wks	-42.02	Yes	**	0.0012
6wks vs. 9wks	-9.667	No	ns	0.3304

Aggrecan				
Tukey's multiple comparisons test	Mean Diff.	Below threshold?	Summary	Adjusted P Value
3wks vs. 6wks	-44.74	Yes	*	0.0247
3wks vs. 9wks	-26.95	No	ns	0.1488
6wks vs. 9wks	17.78	No	ns	0.3747

Tenascin-R				
Tukey's multiple comparisons test	Mean Diff.	Below threshold?	Summary	Adjusted P Value
3wks vs. 6wks	-54.97	Yes	*	0.0204
3wks vs. 9wks	-71.94	Yes	**	0.0059
6wks vs. 9wks	-16.97	No	ns	0.5056

Table S1: Summary of statistical analyses comparing the proportion of β-III tubulin–positive MNs co-expressing PNN components (aggrecan, hyaluronan, or tenascin-R) in 3-, 6-, and 9-week co-cultures of wild-type ESC-derived MNs and astrocytes. One-way ANOVA with Tukey's multiple comparison tests. N=3, 5 images/N.

Tenascin-R					
	Tukey's multiple comparisons test	Mean Diff.	Below threshold?	Summary	Adjusted P Value
3 weeks	WT vs. C9-3	-7.556	No	ns	0.9864
	WT vs. TDP-43	5.25	No	ns	0.9953
	WT vs. Iso-TDP-43	-14.75	No	ns	0.9114
	C9-3 vs. TDP-43	12.81	No	ns	0.9394
	C9-3 vs. Iso-TDP-43	-7.194	No	ns	0.9882
	TDP-43 vs. Iso-TDP-43	-20	No	ns	0.8083
6 weeks	WT vs. C9-3	16.37	No	ns	0.8837
	WT vs. TDP-43	6.111	No	ns	0.9927
	WT vs. Iso-TDP-43	1	No	ns	>0.9999
	C9-3 vs. TDP-43	-10.25	No	ns	0.9674
	C9-3 vs. Iso-TDP-43	-15.37	No	ns	0.9013
	TDP-43 vs. Iso-TDP-43	-5.111	No	ns	0.9957
9 weeks	WT vs. C9-3	24.49	No	ns	0.6964
	WT vs. TDP-43	32.02	No	ns	0.4935
	WT vs. Iso-TDP-43	16.97	No	ns	0.8723
	C9-3 vs. TDP-43	7.526	No	ns	0.9866
	C9-3 vs. Iso-TDP-43	-7.519	No	ns	0.9866
	TDP-43 vs. Iso-TDP-43	-15.04	No	ns	0.9066

Hyaluronan					
	Tukey's multiple comparisons test	Mean Diff.	Below threshold?	Summary	Adjusted P Value
3 weeks	WT vs. C9-3	24.43	No	ns	0.1644
	WT vs. TDP-43	2.399	No	ns	0.9965
	WT vs. Iso-TDP-43	4.315	No	ns	0.9807
	C9-3 vs. TDP-43	-22.03	No	ns	0.2368
	C9-3 vs. Iso-TDP-43	-20.11	No	ns	0.3092
	TDP-43 vs. Iso-TDP-43	1.915	No	ns	0.9982
6 weeks	WT vs. C9-3	12.4	No	ns	0.6962
	WT vs. TDP-43	0.05555	No	ns	>0.9999
	WT vs. Iso-TDP-43	-4.944	No	ns	0.9716
	C9-3 vs. TDP-43	-12.34	No	ns	0.6991
	C9-3 vs. Iso-TDP-43	-17.34	No	ns	0.4355
	TDP-43 vs. Iso-TDP-43	-5	No	ns	0.9706
9 weeks	WT vs. C9-3	5	No	ns	0.9706
	WT vs. TDP-43	1.905	No	ns	0.9983
	WT vs. Iso-TDP-43	-2	No	ns	0.998
	C9-3 vs. TDP-43	-3.095	No	ns	0.9927
	C9-3 vs. Iso-TDP-43	-7	No	ns	0.9253
	TDP-43 vs. Iso-TDP-43	-3.905	No	ns	0.9856

Aggrecan					
	Tukey's multiple comparisons test	Mean Diff.	Below threshold?	Summary	Adjusted P Value
3 weeks	WT vs. C9-3	-23.45	No	ns	0.7057
	WT vs. TDP-43	-10.89	No	ns	0.9581
	WT vs. Iso-TDP-43	-10.29	No	ns	0.9643
	C9-3 vs. TDP-43	12.56	No	ns	0.9378
	C9-3 vs. Iso-TDP-43	13.17	No	ns	0.9294
	TDP-43 vs. Iso-TDP-43	0.6032	No	ns	>0.9999
6 weeks	WT vs. C9-3	-0.06611	No	ns	>0.9999
	WT vs. TDP-43	7.414	No	ns	0.986
	WT vs. Iso-TDP-43	8.486	No	ns	0.9794
	C9-3 vs. TDP-43	7.48	No	ns	0.9857
	C9-3 vs. Iso-TDP-43	8.552	No	ns	0.9789
	TDP-43 vs. Iso-TDP-43	1.072	No	ns	>0.9999
9 weeks	WT vs. C9-3	19.75	No	ns	0.8008
	WT vs. TDP-43	5.635	No	ns	0.9937
	WT vs. Iso-TDP-43	-10.67	No	ns	0.9605
	C9-3 vs. TDP-43	-14.11	No	ns	0.9149
	C9-3 vs. Iso-TDP-43	-30.41	No	ns	0.5125
	TDP-43 vs. Iso-TDP-43	-16.3	No	ns	0.876

Table S2: Statistical summary of PNN-like deposition assessed by immunocytochemistry in iPSC-derived and wild-type ESC-derived MNs lines following 3, 6, or 9 weeks of co-culture. The percentage of double-positive MNs (β-III tubulin and either hyaluronan, tenascin-R, or aggrecan) was quantified from 5 images per biological replicate (N = 3). Statistical significance was assessed using one-way ANOVA with Tukey's multiple comparisons test.

CHAT				
Tukey's multiple comparisons test	Mean Diff.	Below threshold?	Summary	Adjusted P Value
WT vs TDP-43	-0.05293	Yes	***	0.0001
C9-3 vs. TDP-43	-0.05723	Yes	****	<0.0001
IsoC9-2 vs. TDP-43	-0.06251	Yes	****	<0.0001
C9-2 vs. TDP-43	-0.05188	Yes	***	0.0002
Iso-TDP-43 vs. TDP-43	-0.04568	Yes	***	0.0006

HAPLN4				
Tukey's multiple comparisons test	Mean Diff.	Below threshold?	Summary	Adjusted P Value
WT vs. TDP-43	-0.06556	Yes	***	0.0003
C9-3 vs. TDP-43	-0.06943	Yes	***	0.0002
IsoC9-2Δ vs. TDP-43	-0.07406	Yes	***	0.0001
C9-2 vs. TDP-43	-0.06539	Yes	***	0.0004
Iso-TDP-43 vs. TDP-43	-0.06403	Yes	***	0.0004

HAS3				
Tukey's multiple comparisons test	Mean Diff.	Below threshold?	Summary	Adjusted P Value
WT vs. TDP-43	-0.1769	Yes	**	0.0014
C9-3 vs. TDP-43	-0.1823	Yes	**	0.0011
IsoC9-2 vs. TDP-43	-0.2068	Yes	***	0.0003
C9-2 vs. TDP-43	-0.1961	Yes	***	0.0006
Iso-TDP-43 vs. TDP-43	-0.1474	Yes	**	0.0061

HYAL2				
Tukey's multiple comparisons test	Mean Diff.	Below threshold?	Summary	Adjusted P Value
WT vs. TDP-43	-0.1421	Yes	**	0.0094
C9-3 vs. TDP-43	-0.1374	Yes	*	0.012
IsoC9-2 vs. TDP-43	-0.1796	Yes	**	0.0015
C9-2 vs. TDP-43	-0.1352	Yes	*	0.0134
Iso-TDP-43 vs. TDP-43	-0.121	Yes	*	0.0279

NCAN				
Tukey's multiple comparisons test	Mean Diff.	Below threshold?	Summary	Adjusted P Value
IsoC9-2 vs. TDP-43	-0.1415	Yes	*	0.0439

PTPRZ1				
Tukey's multiple comparisons test	Mean Diff.	Below threshold?	Summary	Adjusted P Value
C9-3 vs. TDP-43	-0.07853	Yes	**	0.0076
IsoC9-2 vs. TDP-43	-0.08429	Yes	**	0.0045
C9-2 vs. TDP-43	-0.08098	Yes	**	0.0061

SEMA3A				
Tukey's multiple comparisons test	Mean Diff.	Below threshold?	Summary	Adjusted P Value
WT vs. TDP-43	-0.3188	Yes	***	0.0008
C9-3 vs. TDP-43	-0.3445	Yes	***	0.0004
IsoC9-2 vs. TDP-43	-0.3796	Yes	***	0.0002
C9-2 vs. TDP-43	-0.2866	Yes	**	0.0021
Iso-TDP-43 vs. TDP-43	-0.2995	Yes	**	0.0014

Table S3: Statistical summary of qPCR analyses performed on hESC- and hiPSC-derived MNs following 3 weeks of co-culture with mouse astrocytes. Only genes showing statistically significant differential expression are reported. Statistical significance was assessed using one-way ANOVA with Tukey's multiple comparisons test, with N = 3 independent differentiations.

Dataset number	GEO ascension number	Details	Species	Age	Disease status	Anatomical area	Direction of comparison
1	E-GEOD_52118	Microarray, laser capture microdissection	Mouse	P7	Healthy/non-ALS	OMN	OMN vs SMN
1	E-GEOD_52118	Microarray, laser capture microdissection	Mouse	P7	Healthy/non-ALS	Onuf's nucleus	Onuf's nucleus vs SMN
2	GSE3343	Microarray	Mouse	10 wks	Healthy/non-ALS	OMN	OMN vs SMN
2	GSE3343	Microarray	Mouse	6 wks	ALS - SOD1	OMN	OMN vs SMN
3	GSE118620	Bulk RNA sequencing	mESC	9 days in culture	Healthy/non-ALS	OMN	OMN vs SMN
4	GSE132972	Bulk RNA sequencing	hiPSC	17 days in culture	ALS - SOD1	OMN	OMN vs SMN
4	GSE132972	Bulk RNA sequencing	hiPSC	17 days in culture	ALS - C9ORF72	OMN	OMN vs SMN
5	E-GEOD-40438	Microarray, laser capture microdissection	Human	Post-mortem, age 59	Healthy/non-ALS	OMN	OMN vs SMN
6	GSE93939	Bulk RNA sequencing; laser capture microdissection	Human	Post-mortem	Healthy/non-ALS	OMN	OMN vs SMN
6	GSE93939	Bulk RNA sequencing; laser capture microdissection	Human	Post-mortem	Healthy/non-ALS	Onuf's nucleus	Onuf's nucleus vs SMN
6	GSE93939	Bulk RNA sequencing; laser capture microdissection	Human	Post-mortem	ALS	OMN	OMN vs SMN

Table S4: Transcriptomic datasets used to compare expression of PNN-associated genes in tissues from regions exhibiting relative vulnerability or resistance to MN deathin ALS. Abbreviations: OMN, oculomotor nerve/cranial nerve III; SMN, spinal cord motor neurons; mESC, mouse embryonic stem cells; P7, postnatal day 7; hiPSC, human induced pluripotent stem cells.

Supplementary Code 1

Macro script written for use in FIJI software to threshold images of PNN staining. Script was applied to original images obtained using a Zeiss LSM 980 confocal microscope.

```
// ask user to select a folder
dir1 = getDirectory("C:/Users/Kerin/Downloads");
// get the list of files (& folders) in it
list = getFileList(dir1);
// prepare a folder to output the images
dir2= dir1 + File.separator + "Overlay and PNN stain" + File.separator ;
File.makeDirectory(dir2);
//setBatchMode(true);
for (i=0; i<list.length; i++) {
showProgress(i+1, list.length);
open(dir1+list[i]);
run("Z Project...", "projection=[Max Intensity]");
title = getTitle();
run("Split Channels");
// Run threshold and apply colour to each z stack image for each channel
title1 = "C1-" + title;
selectWindow(title1); {
setAutoThreshold("Default dark");
//run("Threshold...");
setThreshold(47, 255);
setOption("BlackBackground", false);
run("Convert to Mask");
//run("Channels Tool...");
```

```
run("Cyan");
//run("Flatten");
// rename("TUJ1");
//TUJ1 = getTitle();
// saveAs("TIFF", output_dir_composite + TUJ1);
}
title2 = "C2-" + title;
selectWindow(title2); {
setAutoThreshold("Default dark");
//run("Threshold...");
setThreshold(50, 255);
setOption("BlackBackground", false);
run("Convert to Mask");
//run("Channels Tool...");
run("Green");
// run("Flatten");
//rename("DAPI");
//DAPI = getTitle();
// saveAs("TIFF", output_dir_composite + DAPI);
}
title3 = "C3-" + title;
selectWindow(title3); {
setAutoThreshold("Default dark");
//run("Threshold...");
setThreshold(50, 255);
setOption("BlackBackground", false);
run("Convert to Mask");
//run("Channels Tool...");
```

```
run("Red");

//rename("Aggrecan or other PNN staining name");

//Aggrecan = getTitle();

//saveAs("TIFF", dir2 + title);

}

// Run overlay

//Here overlay DAPI on TUj1 first and then add ACAN over DAPI/TUj image

selectWindow(title3);

saveAs("TIFF", dir2 + title3);

selectWindow(title1);

run("Add Image...", "image=[&title2] x=0 y=0 opacity=50 zero");

rename("TUJ_DAPI" + title1);

run("Flatten");

saveAs("TIFF", dir2 + "TUJ_DAPI" + title1);

run("Add Image...", "image=[&title3] x=0 y=0 opacity=50 zero");

rename("W_PNN_Stain" + title1);

run("Flatten");

saveAs("TIFF", dir2 + "W_PNN_Stain" + title1);

}

run("Close All");
```

Supplementary Code 2

Macro script written for use in FIJI software to threshold images of TDP-43 staining in all cell lines. Script was applied to original images obtained using Zeiss LSM 980 confocal microscope.

```
// ask user to select a folder

dir = getDirectory("C:/Users/Kerin/Desktop/Figures/PNN counts");

// get the list of files (& folders) in it

fileList = getFileList(dir);

// prepare a folder to output the images

output_dir_splits = dir + File.separator + " output_splits_Threshold" + File.separator ;

File.makeDirectory(output_dir_splits);

//activate batch mode

setBatchMode(true);

// LOOP to process the list of files

for (i = 0; i < lengthOf(fileList); i++) {

// define the "path"

// by concatenation of dir and the i element of the array fileList

current_imagePath = dir+fileList[i];

// check that the currentFile is not a directory

if (!File.isDirectory(current_imagePath)){

// open the image and split

open(current_imagePath);

// get some info about the image

getDimensions(width, height, channels, slices, frames);

//setAutoThreshold("Default");

run("Threshold...");

setThreshold(0, 30);
```

```
setOption("BlackBackground", false);

run("Convert to Mask");

title = getTitle();

saveAs("tiff", output_dir_splits +title);

}

// make sure to close every images befores opening the next one

run("Close All");

}

setBatchMode(false);
```
