# Supplementary material for: ALS mutations do not alter perineuronal net formation in human stem cell-derived motor neurons
Source: Commun Biol. 2026 May 19;9:1062. doi: 10.1038/s42003-026-10244-6 (PMC13454605; doi:10.1038/s42003-026-10244-6)
Supplement: Supplementary file 2 [file 42003_2026_10244_MOESM3_ESM.pdf]

Description of Additional Supplementary Files:

File name: Supplementary Data 1

Description: The source data behind the graphs in the paper
