# Supplementary material for: ALS mutations do not alter perineuronal net formation in human stem cell-derived motor neurons
Source: Commun Biol. 2026 May 19;9:1062. doi: 10.1038/s42003-026-10244-6 (PMC13454605; doi:10.1038/s42003-026-10244-6)
Supplement: Supplementary file 3 [file 42003_2026_10244_MOESM4_ESM.pdf]

nature portfolio

Corresponding author(s): Eileen GENTLEMAN

Last updated by author(s): 30/09/2025

Reporting Summary

Nature Portfolio wishes to improve the reproducibility of the work that we publish. This form provides structure for consistency and transparency in reporting. For further information on Nature Portfolio policies, see our Editorial Policies and the Editorial Policy Checklist.

Statistics

For all statistical analyses, confirm that the following items are present in the figure legend, table legend, main text, or Methods section.

n/a	Confirmed	
☐	☒	The exact sample size (*n*) for each experimental group/condition, given as a discrete number and unit of measurement
☐	☒	A statement on whether measurements were taken from distinct samples or whether the same sample was measured repeatedly
☐	☒	The statistical test(s) used AND whether they are one- or two-sided<br>*Only common tests should be described solely by name; describe more complex techniques in the Methods section.*
☒	☐	A description of all covariates tested
☐	☒	A description of any assumptions or corrections, such as tests of normality and adjustment for multiple comparisons
☐	☒	A full description of the statistical parameters including central tendency (e.g. means) or other basic estimates (e.g. regression coefficient) AND variation (e.g. standard deviation) or associated estimates of uncertainty (e.g. confidence intervals)
☐	☒	For null hypothesis testing, the test statistic (e.g. *F*, *t*, *r*) with confidence intervals, effect sizes, degrees of freedom and *P* value noted<br>*Give P values as exact values whenever suitable.*
☒	☐	For Bayesian analysis, information on the choice of priors and Markov chain Monte Carlo settings
☒	☐	For hierarchical and complex designs, identification of the appropriate level for tests and full reporting of outcomes
☒	☐	Estimates of effect sizes (e.g. Cohen's *d*, Pearson's *r*), indicating how they were calculated

Our web collection on statistics for biologists contains articles on many of the points above.

Software and code

Policy information about availability of computer code

Data collection	GSE115130/ GSE93939/ E-GEOD-40438/ GSE132972/ GSE3305/ GSE118620/ GSE3343/ E-GEOD_52118
Data analysis	Data were analysed using Graphpad Prism, Version 10 (Graphpad Software, San Diego, California USA).<br>Where noted, some images were analysed using FIJI software and custom code, which is provided in the supplementary notes. R studio version used was 4.4.1 and FIJI version 2.9.

For manuscripts utilizing custom algorithms or software that are central to the research but not yet described in published literature, software must be made available to editors and reviewers. We strongly encourage code deposition in a community repository (e.g. GitHub). See the Nature Portfolio guidelines for submitting code & software for further information.

Data

Policy information about availability of data

All manuscripts must include a data availability statement. This statement should provide the following information, where applicable:

- Accession codes, unique identifiers, or web links for publicly available datasets
- A description of any restrictions on data availability
- For clinical datasets or third party data, please ensure that the statement adheres to our policy

Materials availability: Cell lines are available on request and through appropriate MTAs.

Data and code availability: Data are either publicly available or are available upon request to the corresponding author. Code are available within the supplementary information included with this manuscript.

Research involving human participants, their data, or biological material

Policy information about studies with human participants or human data. See also policy information about sex, gender (identity/presentation), and sexual orientation and race, ethnicity and racism.

Reporting on sex and gender	N/A
Reporting on race, ethnicity, or other socially relevant groupings	N/A
Population characteristics	N/A
Recruitment	N/A
Ethics oversight	N/A

Note that full information on the approval of the study protocol must also be provided in the manuscript.

Field-specific reporting

Please select the one below that is the best fit for your research. If you are not sure, read the appropriate sections before making your selection.

☒ Life sciences ☐ Behavioural & social sciences ☐ Ecological, evolutionary & environmental sciences

For a reference copy of the document with all sections, see nature.com/documents/nr-reporting-summary-flat.pdf

Life sciences study design

All studies must disclose on these points even when the disclosure is negative.

Sample size	Sample size calculations were not explicitly performed.
Data exclusions	No data were excluded from analyses.
Replication	All images and data represent a minimum of 3 biological replicates and are described in detail in the manuscript.
Randomization	There was no randomisation in this study.
Blinding	Blinding was not possible and was not relevant to the study.

Reporting for specific materials, systems and methods

We require information from authors about some types of materials, experimental systems and methods used in many studies. Here, indicate whether each material, system or method listed is relevant to your study. If you are not sure if a list item applies to your research, read the appropriate section before selecting a response.

Materials & experimental systems

n/a	Involved in the study	
☐	☒	Antibodies
☐	☒	Eukaryotic cell lines
☒	☐	Palaeontology and archaeology
☒	☐	Animals and other organisms
☒	☐	Clinical data
☒	☐	Dual use research of concern
☒	☐	Plants

Methods

n/a	Involved in the study	
☒	☐	ChIP-seq
☒	☐	Flow cytometry
☒	☐	MRI-based neuroimaging

Antibodies

Antibodies used	Aggrecan core protein bs-1223R-BSS Rabbit Bioss 1:200

Antibodies used	Hyaluronic acid binding protein 385911 Biotinylated Sigma Aldrich 1:200<br>hTenascin R pAb AF3865-SP Goat RnD Systems 1:200<br>Wisteria floribunda agglutinin L1516 Biotinylated Sigma Aldrich 1:200<br>Beta-III tubulin MAB1195 Mouse RnD Systems 1:1000<br>Glial acidic fibrillary protein 2.2B10 Rat Thermo Fisher 1:250<br>TDP-43 107-82-2-AP Rabbit Protein Tech 1:300<br>Streptavidin AlexaFluor™ 647 S32357 Abcam 1:1000<br>Donkey anti-Mouse IgG, AlexaFluor™ 488 A-21202 Donkey Thermo Fisher 1:1000<br>Donkey anti- Rabbit IgG, AlexaFluor™ 568 A10042 Donkey Thermo Fisher 1:1000<br>Donkey anti-Goat IgG, AlexaFluor™ 568 A-11057 Donkey Thermo Fisher 1:1000<br>Donkey anti-Rat IgG, AlexaFluor™ 568 A78946 Donkey Thermo Fisher 1:1000<br>Donkey anti-Rat IgG, AlexaFluor™ 647 A48272 Donkey Thermo Fisher 1:1000<br>Streptavidin AlexaFluor™ 647 S32357 Abcam 1:1000<br>Donkey anti-Mouse IgG2a, AlexaFluor™ 647 A-21241 Goat Thermo Fisher 1:1000
Validation	All antibodies were commercially available, validated and suitable for use with human cells, as specified by the manufacturer.

Eukaryotic cell lines

Policy information about cell lines and Sex and Gender in Research

Cell line source(s)	H9 human ESC line (Wildtype, WT) WiCell (female)<br>M211R2 C9orf72-mutant human iPSC line (C9-3) Mehta et al. Acta Neuropathol 202146 (male)<br>DN1qV4 C9orf72-mutant human iPSC line (C9-2) Mehta et al. Acta Neuropathol 202146 (male)<br>Isogenic DN1qV4 control human iPSC line (Iso-C9-2) Mehta et al. Acta Neuropathol 202146 (male)<br>TDP-43 G298S-mutant human iPSC line (TDP-43-G298S) Barton et al. Brain Commun 202147 (male)<br>Isogenic TDP-43 G298S control human iPSC line (Iso-TDP-43-G298S) Harley et al. Cell Rep 202336 (male)<br>H9 human ESC line, Wildtype, WT, Hb9::CD14 transgenic Harley et al. Cell Rep 202336 (female)<br>M211R2 C9orf72-mutant human iPSC line C9-3, Hb9::CD14 transgenic Harley et al. Cell Rep 202336 (male)<br>DN1qV4 C9orf72-mutant human iPSC line, C9-2, Hb9::CD14 transgenic This study (male)<br>Isogenic DN1qV4 control human iPSC line, Iso-C9-2, Hb9::CD14 transgenic This study (male)<br>TDP-43 G298S-mutant human iPSC line, TDP-43-G298S, Hb9::CD14 transgenic Harley et al. Cell Rep 202336 (male)<br>Isogenic TDP-43 G298S control human iPSC line, Iso-TDP-43-G298S, Hb9:CD14 transgenic Harley et al. Cell Rep 202336 (male)<br>H9 human ESC line, Wildtype, Hb9::CD14/CAG::CAG::ChR2-YFP transgenic Harley et al. Cell Rep 202336 (male)<br>M211R2 C9orf72-mutant human iPSC line C9-3, Hb9::CD14/CAG::CAG::ChR2-YFP transgenic Harley et al. Cell Rep 202336 (male)<br>TDP-43 G298S-mutant human iPSC line TDP-43-G298S, Hb9::CD14/CAG::CAG::ChR2-YFP transgenic Harley et al. Cell Rep 202336 (male)<br>Isogenic TDP-43 G298S control human iPSC line Iso-TDP-43-G298S, Hb9::CD14/CAG::CAG::ChR2-YFP transgenic Harley et al. Cell Rep 202336 (male)<br>IB10 mouse embryonic stem cell (mESC) line, GFAP::CD14/CAG:: GDNF transgenic Bryson et al., Science 201435 (male)
Authentication	All iPSC lines were checked for pluripotency and differentiation to the three germ layers by qPCR.
Mycoplasma contamination	All cell lines were routinely tested and confirmed to be negative for mycoplasma.
Commonly misidentified lines<br>(See ICLAC register)	No misidentified cell lines were used.

Plants

Seed stocks	*Report on the source of all seed stocks or other plant material used. If applicable, state the seed stock centre and catalogue number. If plant specimens were collected from the field, describe the collection location, date and sampling procedures.*
Novel plant genotypes	*Describe the methods by which all novel plant genotypes were produced. This includes those generated by transgenic approaches, gene editing, chemical/radiation-based mutagenesis and hybridization. For transgenic lines, describe the transformation method, the number of independent lines analyzed and the generation upon which experiments were performed. For gene-edited lines, describe the editor used, the endogenous sequence targeted for editing, the targeting guide RNA sequence (if applicable) and how the editor was applied.*
Authentication	*Describe any authentication procedures for each seed stock used or novel genotype generated. Describe any experiments used to assess the effect of a mutation and, where applicable, how potential secondary effects (e.g. second site T-DNA insertions, mosiacism, off-target gene editing) were examined.*
